# Mineralochemical Mechanism for the Formation of Salt Volcanoes: The Case of Mount Dallol (Afar Triangle, Ethiopia)

**DOI:** 10.1021/acsearthspacechem.2c00075

**Published:** 2022-10-19

**Authors:** Fermín Otálora, Fernando Palero, Evgenia-Maria Papaslioti, Juan Manuel García-Ruiz

**Affiliations:** Laboratorio de Estudios Cristalográficos. Instituto Andaluz de Ciencias de la Tierra, CSIC-Universidad de Granada, Armilla, Granada18100, Spain

**Keywords:** Dallol, mineral dehydration, salt volcano, Houston formation, geothermal model

## Abstract

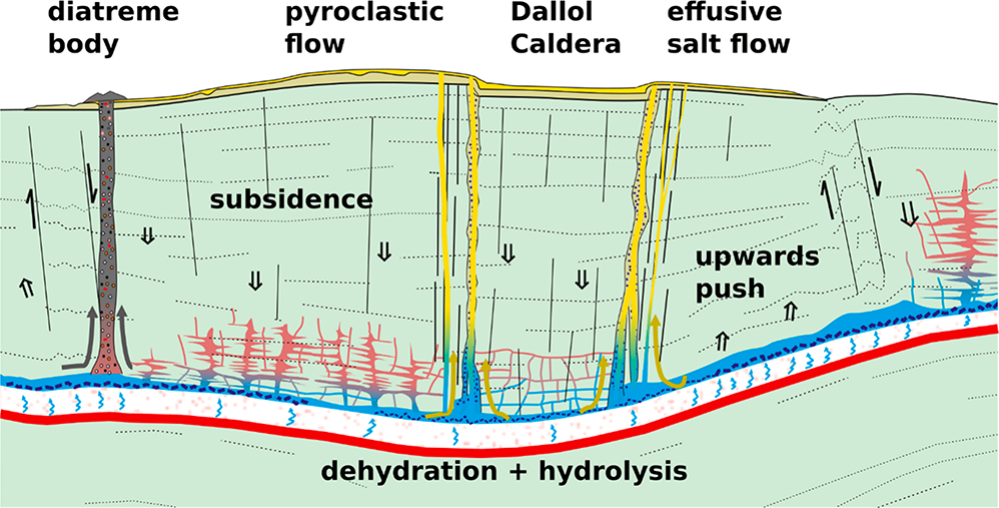

A genetic model is proposed for the formation and evolution of volcano-like structures from materials other than molten silicate rocks. The model is based on Mount Dallol (Afar Triangle, Ethiopia), currently hosting a conspicuous hydrothermal system with hot, hyper-acidic springs, forming a colorful landscape of unique mineral patterns. We reason that Mount Dallol is the last stage of the formation of a salt volcano driven by the destabilization of a thick sequence of hydrated minerals (the Houston Formation) after the emplacement of an igneous intrusion beneath the thick Danakil evaporitic sequence. Our claim is supported by field studies, calculations of the mineral/water volume balance upon mineral dehydration, and by a geothermal model of the Danakil basin predicting a temperature up to 220 °C at the Houston Formation after the intrusion of a basaltic magma without direct contact with the evaporitic sequence. Although insufficient for salt melting, this heating triggers mineral dehydration and hydrolysis, leading to a total volume increase of at least 25%. The released brine is segregated upward into a pressurized chamber, where the excess volume produced the doming of Mount Dallol. Later, the collapse of the dome formed a caldera and the emission of clastic flows. The resulting structures and materials resemble volcanic lava flows in distribution, structure, and texture but are entirely made of salty materials. This novel mechanism of the generation of pressurized brines and their later eruption extends the relevance of volcanologic studies to lower temperature ranges and unanticipated geologic contexts on Earth and possibly also on other planets.

## 1 Introduction

The Afar Triangle in the far north of the East African Rift System constitutes a paradigmatic example of the evolution of an active continental rifting, separating Africa from Arabia (e.g., refs (1 and 2)). This rift evolution led to the 300 km wide Afar depression, located at the intersection of the Red Sea, the Gulf of Aden, and the East African Rift, known as the Afar triple junction (e.g., refs (1, 3, and 4)). At the north of this depression is the Danakil, a deserted salt plain of 4000 km^2^ massive marine evaporitic deposits formed when the region was connected at the north to the Red Sea^5^ (Figure S1). Beyond the geothermal and mining potential of this area and its interest in continental breakup studies, its geological importance has recently increased because of the discovery of a unique polyextreme hydrothermal field on the top of an elevation known as Mount Dallol. Understanding this geological site is a challenge of great interest due to its extreme geochemistry, its intriguing mineral patterns formed by purely inorganic processes, and its significance to the investigation of the limits of life.^6−8^

Mount Dallol stands on top of the 2.2 km thick Danakil evaporitic sequence,^9,10^ deposited after the Danakil Depression was recurrently flooded by seawater through the Gulf of Zula (Eritrea) during late Tertiary and most of the Quaternary.^11,12^ The stratigraphy of the central part of the Danakil depression is difficult to be studied due to the absence of adequate outcrops^13^ but has been investigated using boreholes and seismic profiles up to a depth of around 1000 m.^2,14^ Away from the basin margin, three large-scale evaporite units have been identified from top to bottom: (1) Upper Rock Salt (URS), (2) the potash-rich Houston Formation, and (3) Lower Rock Salt (LRS).^2^ The LRS, 500–1000 m in thickness, is composed of coarsely crystalline halite beds that alternate with anhydrite. It was deposited during a first flooding episode that ended 240,000 years ago.^2^ The Houston Formation, which is key for our model, is a shallower potash layer deposited on top of the LRS.^14^ A second flooding period occurred 120,000 years ago,^15,16^ producing the URS, consisting of another 1 km thick layer of halite with some anhydrite and shales,^2,17^ with the latter being almost completely absent in the LRS. The evaporitic sedimentation ended around 33,500 years ago, when the emergence of a new volcano, Mount Alid (NE Dallol), closed the basin at the North.^18,19^ The Houston Formation, situated between the LRS and the URS, is composed of four members,^14^ from the base to the top: the kainite member (∼8 m), the intermediate member formed by carnallite and bischofite (∼53 m), a layer of sylvite-halite-anhydrite (∼5.5 m), and a halite-anhydrite member (“Marker Beds”; ∼8 m). The intermediate member contains a thick layer (∼45 m) of bischofite (MgCl_2_·6H_2_O) sandwiched between two thinner layers of carnallite. The minerals present in the original, unaltered formation are the following (in the order of increasing abundance): bischofite, carnallite, kainite, kieserite, and minor amounts of anhydrous minerals (mainly halite, sylvite, and anhydrite).^14,20^

The Danakil depression is characterized by active basaltic volcanism fed by relatively shallow-level (1–2 km deep) magma reservoirs, especially in the Erta Ale region, 80 km SE of Dallol.^21−23^ The origin of Mount Dallol has been assigned to this volcanic activity,^23^ but neither the dome materials nor the seismic profiles under Dallol support the role of igneous effusive processes in its formation. No volcanic products (lava, ash-fall, or scoria) are found on the surface, near it, or in deep boreholes except occasionally minor, localized tuff/ash-fall deposits in the last 200 m or a basalt/dolerite level found at 170 m depth in one of the boreholes.^2^ These occasional volcanic materials have been consistently interpreted to be related to eruptions at Erte Ale or less often, localized lava flows.^2^ Even rocks associated with a recent phreatic crater formed in 1926 at the SW border of Dallol (Black Mountain)^24^ are devoid of igneous materials. Seismic reflection profiles, acquired in 2010 by TESLA-IMC, show the more prominent stratigraphic surfaces and normal faults under Dallol.^2^ These profiles show no indication of the presence of igneous materials in the shallow subsurface. Ongoing magma intrusions are expected in this rifting context, and evidence of the intrusion of a 9 km long, 0.06 km^3^ dike,^23^ have been obtained from radar interferometry data. The earthquake associated with this intrusion was located around 13 km SE of Dallol. Still, no large intrusions have been reported so far above the shallow magma chamber located 2.4 km beneath Dallol.^23^ This is consistent with results on the extension of the Danakil basin:^2^ the fault magnitude, slip, and amount, along with the geometry of the basin, suggest that fault-related extension is dominant (at least in the North) rather than dike intrusion extension. The lack of near-surface igneous emplacements or extrusive igneous rocks on or around Dallol does not mean that the underlying magmatic materials are irrelevant to the origin of Mount Dallol, which has been reported to be a salt dome, generated by the migration and stalling of molten magma at depth.^16,20,21^ Still, many features found in the area, such as craters, clastic flows (Figure S2), fluid materials flow (Figure S3), explosive breccias, and diatreme bodies (Figure S4), are characteristic of volcanic processes. The puzzling feature of these structures is that they are made of salty materials without the presence of igneous rocks. The mechanisms leading to the formation of volcanic-like salt structures, and even of large-scale halo-volcano edifices, are not yet understood. Recent studies^13^ show these features and explain their presence with eruptive melted salts produced by magma/evaporite interactions. Still, the combined absence of igneous rocks and the lack of evidence for large magmatic emplacements above 2.4 km suggest the need for a mechanism explaining these eruptive/explosive features. In this study, we present a novel mechanism that can explain the development of structures and edifices sharing many features with ordinary volcanoes but entirely made of salt.

This mechanism is controlled by the migration, effusion, and solidification of hot, viscous slurries of salty brines that reach the surface from a pressurized fluid chamber, where brine accumulates due to dehydration reactions. This proposal is based on (a) our field observations of the volcanic-like structures in Mount Dallol during field campaigns in 2017 and 2018 and the analysis of the collected samples (Section 3.1), (b) a model for the thermal evolution of the Danakil evaporitic sequence after a magmatic intrusion (Section 3.2), and (c) a feasible mineralogical route for the generation of pressurized fluids by thermal destabilization of hydrated evaporites (Section 3.3). Combining these results allowed us to build a self-consistent framework that explains the origin and the evolution of Mount Dallol to date, including the hyper-acidic chemistry of the current hydrothermal system.

Our model shares some processes and outcomes with other volcano-building processes, such as ice or mud volcanoes^25,26^ that are the most widespread. Mud volcanism is currently recognized as the result of buoyancy-driven upward migration of fluidized materials originated in a sedimentary sequence by burial, tectonic, thermogenic, or biogenic processes, leading to overpressure at a given depth under the sedimentary sequence.

## 2 Methods

The distribution of the lithologic units in Dallol and the nearby sites were studied during three field campaigns (2016, 2017, and 2019) resulting in a detailed geological description, recorded as a geologic map, using a 0.75m resolution satellite image (January 2019, Pleiades CNES – Distribution Airbus DS) as the cartographic base. This geological study was complemented with underground information from published seismic profiles by mining companies.^2,27^ The mineralogy of the collected solid samples was determined by powder X-ray diffraction (XRD), using a PANalytical MPD diffractometer with a Bragg–Brentano parafocusing geometry and Cu Kα radiation (operating at 40 mA and 45 kV) at the Andalusian Institute of Earth Sciences (IACT; Granada, Spain). Instrument configuration included programmable divergence slits in the incident and diffracted beams, placement of a 0.25° fix antiscatter slit in the incident path and a PSD detector PIXel. Data processing was conducted using the software HighScore Plus from PANalytical X’Pert PRO (mineral database: Pdf2HSP/PAN-ICSD). Complementary analysis of mineral phases was carried out via a field-emission scanning electron microscope with an AURIGA system (FESEM; Zeiss Supra 40Vp), coupled with an energy dispersive X-ray spectrometer (EDS), also connected to a Renishaw inVia Raman spectrometer fitted with a Nd:YAG 532 nm laser and a near-infrared diode 785 nm laser, with maximum powers of 500 and 100 mW, respectively, at the Instrumentation Centre of the University of Granada, Spain. Thin sections of selected samples were also observed by polarized light microscopy, using a NIKON AZ100 optical microscope coupled to a JENOPTIK ProgRes CT3 camera.

Water/mineral volume balances have been computed using published thermogravimetric data (TGA) of bischofite^28^ and carnallite^29^ thermal transformations. These studies were used to build a quantitative model for the temperature-dependent volume of the different mineral phases involved and that of the released water as a function of temperature during dehydration. Unfortunately, data on phase relations^30^ is incomplete and the properties of high T, saturated solutions are not known accurately. These deficiencies were overcome by using experimental TGA data on the dehydration reactions. The derivative of the TGA profile was fitted to the sum of four (bischofite) and two (carnallite) exponential-Gaussian hybrid distributions so that the integration of each one separately allowed us to compute the volume variations during the thermal dehydration reactions. The molar volume of all phases was computed using published crystallographic data, mainly from the American Mineralogist Crystal Structure Database.^31^ PT-dependent density of water values and computed water values, computed using the IAPWS95 library^32^ were used to calculate the liquid fraction.

A finite differences code was implemented to calculate the temperature gradient at the Danakil graben under Dallol. This code used constant-temperature boundaries at the intrusion (1200 °C) and the surface of Mount Dallol (35 °C, mean yearly air temperature) and periodic boundaries sideways. Cooling of the intrusion and crystallization of igneous minerals is neglected because, in a large magmatic body, they operate at a time-scale much longer than that in our model. The finite differences schema used and the parameters to compute the lithology-dependent thermal properties were taken from a previous work.^33^ The code was used to compute the transient evolution from the time of the intrusion to 250,0000 years later, when a steady gradient was established. All calculations have been implemented using Python 3^34^ code calling the NumPy numerical library^35^ for high-performance vectorized calculations. A detailed description of the methodology is included in the Supporting Information.

## 3 Results

### 3.1 Lithology and structures

**Figure 1:**
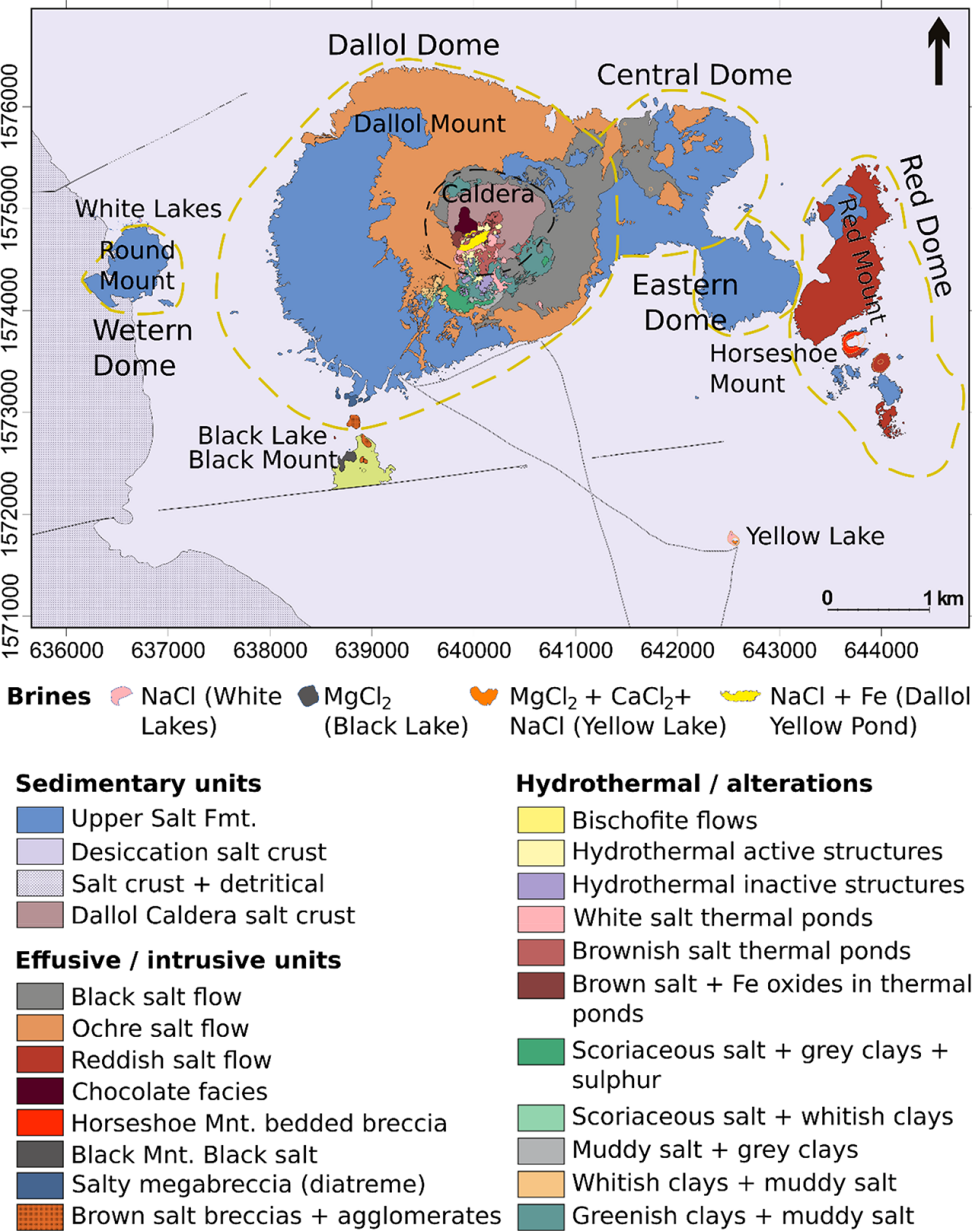
Geological map of the study area.

**Figure 2:**
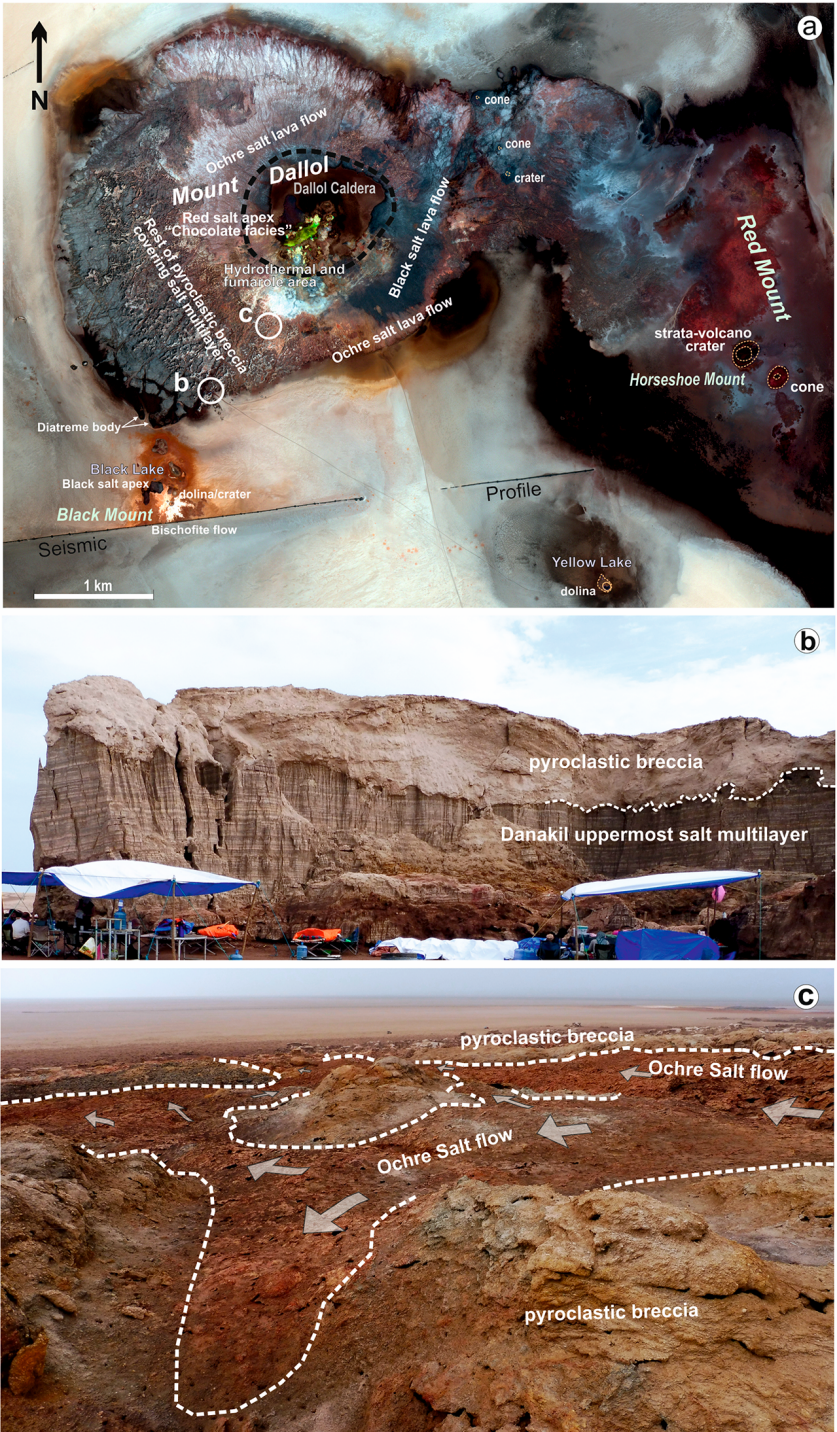
Views of the volcanic-like features of Dallol. (a) Satelite image of Dallol showing the typical volcanic landscape of the area (Pléiades Imagery, CNES – distribution Airbus DS). The places and features mentioned in the text are labeled. Some typical “volcanic” features (craters, cones, caldera, pyroclastic deposits, hydrothermal fields, etc.) are pointed out. Circles indicate the areas shown in photos b and c. (b) Outcrop of the clastic breccia on top of the Danakil uppermost salt unit by the campsite at the SW Dallol dome. The unconforming contact at an old erosive surface on the underlying evaporitic sequence, with typical dissolution forms is highlighted (dashed line to the right). (c) Perspective view of the ochre salt flows in the Southern slope of the Dallol dome, where flow of dirty salt was channelled on the previously eroded pyroclastic breccia. The limits of the flow and the flow directions are highlighted.

Figure 1 summarizes our field observations about the effusive units described below, their relation with the unaffected sedimentary units of the Danakil salt plain, and the location of the current hydrothermal deposits, including the current chemical nature of the ponds studied. Mount Dallol is a dome structure, 2.5–3.5 km in diameter, with a circular depression in the center (Figure 2a) that is currently a place of intense hydrothermal activity. Morphologically, it looks like a typical volcano, and morphostructures reminiscent of volcanic terrains are readily observed: domes, craters, clastic flows (Figure S2), fluid materials flow (Figure S3), explosive breccias, and diatreme bodies^36^ (Figure S4), all of them made of salt. Several lithological units, different from those in the underlying sedimentary sequence and showing features typical of volcanic rocks, have been identified above the sedimentary materials of the dome during our field campaigns.

An unusual breccia layer made of anhydrite, lutite, and shale fragments with a clay matrix is widely observed in the dome area directly above the evaporitic materials of the Upper Salt Unit (Figure 2b and Figure S2). The minerals identified by whole-rock X-ray diffraction analysis are (from major to minor) anhydrite, jarosite, halite, calcite, quartz, natrojarosite, and barite. The occurrence of this breccia is restricted to the dome and the nearest surrounding areas. This up to 7 m thick layer, currently observed as isolated patches due to erosion, was interpreted as the concordant end of this sedimentary unit.^13,15,30^ Nevertheless, our field observations show that the breccias have an unconformable contact, and they were deposited on the irregular surface of the underlying halite layers (Figure 2b and Figure S2a). This contact can only be interpreted as a result of the erosion/dissolution of these salty materials prior to the deposition of breccias, which are sealing a paleorelief. Therefore, the Dallol dome had to be already elevated at the time of deposition of those breccias. Two different levels have been identified within this breccia: (i) a lower one with 1–2 m of disordered breccia made of banded anhydrite blocks (up to 30 cm size), shales, and lutites with a rough size classification but no bedding (Figure S2b) and (ii) an upper level of up to 5 m thickness, made of the same materials but more rich in clayey matrix; this upper level shows single or double classification that clearly define a layering with no net contacts between beds (Figure S2c), parallel and cross stratification, and slump folding. This bedding has short lateral extension changing quickly to unclassified material. Noticeably, the amount of halite in the breccia is much lower than in other units in the area. Altogether, these breccias show the typical structural and textural characteristics of pyroclastic deposits,^37^ although they are not made of volcanic rocks.

Most of the dome surface is covered by layers of massive salt with light-brown color (ochre salt). These materials appear around the crater rim, particularly at the North and South sides, and probably at the East, where it would be covered by a later dark-gray salt layer (black salt). These layers show the typical features of a “lava flow”: scoria fragments forming a crust at the rock–air surface, drainage tubes, and channeling of flows on talwegs eroded in the underlying materials (typically the breccia; Figure 2c). The black salt is also channeled through talwegs as a flow forming little cones close to the border of the crater (Figure S3a) and infills dikes in the east slope, which are roughly aligned as circles around the rim (Figure S3b,d).

A third type of dark brown-reddish, massive, and homogeneous salt has been observed inside the crater, named “Chocolate facies”, as it resembles a chocolate bar. On the surface, it shows a polygonal structure reminiscent of retraction cracks, that are highlighted due to rainwater dissolution of the interspace between the polygons (Figure S3e). These materials are more compact than the ochre and black salts, and the presence of polygonal diaclases points to slow cooling of a thick body with plastic behavior. Their location is restricted inside the crater, with contacts that seem sharp and vertical but are difficult to observe due to the later dissolution/precipitation of salt. These formations are composed of halite, anhydrite, jarosite, and hematite, as indicated by XRD analysis, in addition to iron phosphate and silvite determined by EDX. Other reddish salt materials have also been observed in the nearby Red Mount but are different from the Chocolate facies. They are more similar to the ochre and black salt layers but richer in clay materials, anhydrite, and lutite fragments from the sedimentary sequence. These materials were not found in the main Dallol dome. Massive black salt similar to these in the Dallol slopes is found in the nearby Black Mount, although forming an intrusive body hosted in the evaporitic sequence, and with properties similar to those of the Chocolate facies. The mineralogy of the ochre, reddish, and black massive salts is very similar, with the differences being in the amount of insoluble materials included and the oxidation state of iron and, probably, other metals present in minor amounts. According to the results of XRD and EDX analyses, the ochre salt includes halite, anhydrite, hematite, magnetite, and jarosite, and the black mountain salt includes halite, magnetite, chromite, jarosite, hematite, magnesium chlorides, barite, and iron phosphates.

Other types of breccias have also been observed in places around the dome. Stratified breccias forming a crater reminiscent of a small stratovolcano are found at the Horseshoe Mount (Figure S4a,b). They contain a very heterogeneous mix of salty materials, both in size (from <1 mm to >1m) and composition (salty materials either from the underlying sedimentary sequence or close to the ochre, black, and reddish massive salts). The overall appearance is that of an explosive-eruptive deposit, with a mixture of many of the pre-existent materials. Megabreccias found in the SW part of the Dallol dome, mainly formed by fragments of the materials from the upper salt evaporitic sequence, share this appearance with a large variation in block size and materials but no stratification (Figure S4c,d). They are reminiscent of diatremic bodies hosted in the evaporitic sequence.

### 3.2 Thermal Evolution of the Danakil Sequence upon Emplacement of a Magmatic Intrusion

**Figure 3:**
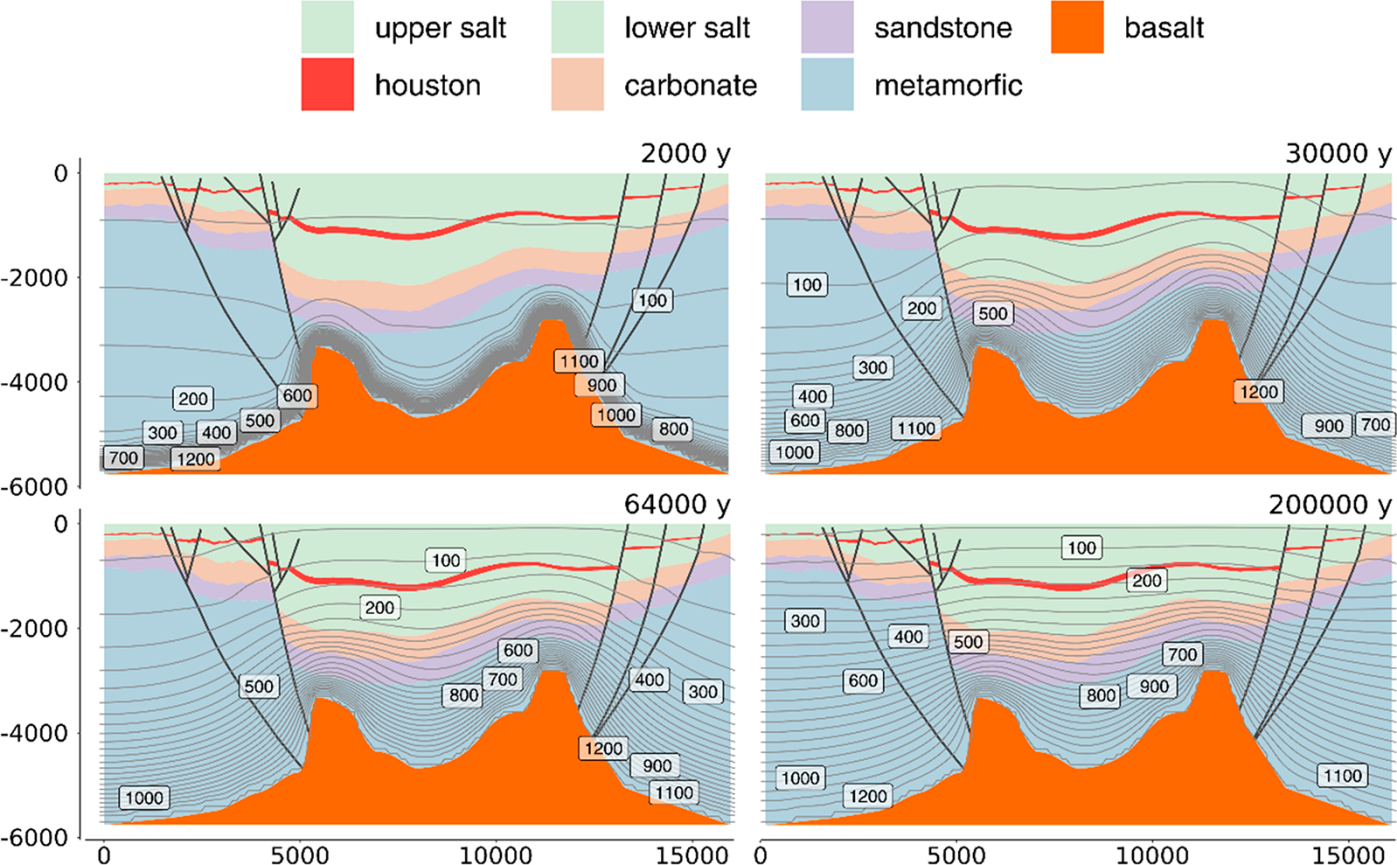
Transient thermal gradient under Dallol after the emplacement of a basaltic body. The four panels show a geological section through the salt units and the Danakil basement along with isothermal lines from 1200 to 50 °C (gray). *t* = 0 corresponds to the intrusion of a basaltic magma body at 1200 °C (orange area at the bottom). Two thousand years later, the intrusion has distorted the thermal gradient, heating areas close to the magma/salt contact. The resulting step gradient gets relaxed over time; the temperature has increased substantially over the central graben after 30,000 years, and the Houston Formation approaches 100 °C. Dehydration reactions start to be very active. After 64,000 years, the Houston Formation reaches a temperature of 150 °C, large enough to produce the dehydration of most of their minerals. Nowadays, the temperature at Houston formation experiences a slow increase toward steady-state, in which it will reach up to 220 °C. Details on the model and a movie of the full evolution are included in the Supporting Information.

The Danakil evaporitic basin is related to extensional faulting and located above a heat source due to magma intrusion in this rift segment.^13^ Based on seismic profiles, the existence of a kind of dike-shaped intrusion located at shallow levels under the western part of Dallol was suggested, fed by a magma chamber located 2.4 km deep.^27^ However, seismic profiles by Allana and Circum Minerals^2^ do not show evidence for the presence of magmatic rocks in the shallow subsurface of Dallol. This means that the heat source affecting the Dallol geothermal area must be located at least 2.5 km deep. To ascertain the impact of such an intrusion on the salt unit, we have calculated the thermal gradient below Mount Dallol and its temporal evolution using a finite difference code implemented by our group (see Section 2 of the Supporting Information for a detailed description of this model). The magma associated with the Rift is basaltic in composition with a temperature of ca. 1200 °C.^38^ Lacking more precise information, the model assumes an irregularly shaped intrusion with a roof-contact at a depth ranging from 5.5 to 2.5 km, consistent with the available seismic profiles.^2,23,27^Figure 3 shows the evolution of the transient geothermal gradient after the emplacement of the magmatic intrusion. The initial steady geothermal gradient is immediately distorted near the intrusion and gets relaxed over time. After 120,000 years, the geothermal gradient is again close to stationary, and the temperature at the level of the Houston Formation has increased to 220 °C. According to these results, the evaporitic sequence, even at the base, was never heated enough to reach a temperature close to the melting point of the mineral phases in the marine sequence (800.7 °C for the major component, halite; Table S1) or even the lower values of their mixtures. Nevertheless, this heating can destabilize most of the hydrated minerals via dehydration and hydrolysis. The Houston Formation reached a temperature compatible with the dehydration of their hydrated minerals (100–150 °C) about 60,000 years after the intrusion, mostly in the center of the graben, where Mount Dallol is located (see position of the respective isotherms in Figure 3c).

### 3.3 Thermal Destabilization of the Hydrated Minerals in the Houston Formation

A hypothesis for the generation and mobilization of pressurized fluids alternative to the melting of salty materials is required due to the lack of direct contact between magmatic and salty materials—except at some minor dikes that could have intruded locally.^23^ We investigated the possibility that these fluids result from the thermal destabilization of hydrated minerals in the metamorphic aureole above the magmatic intrusion and without direct magma-salt contacts.

The more abundant phase in the Houston Formation, bischofite (MgCl_2_·6H_2_O), accounts for 45% of the total volume of the formation. It belongs to the series of hydrated magnesium chlorides, MgCl_2_·*n*H_2_O, *n* = 1, 2, 4, 6, 8, 12, with *n* = 6 (bischofite) being the most fully hydrated of these mineral phases at room temperature.^39^ As temperature increases, these hydrated phases decompose according to the following reactions:
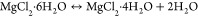
1
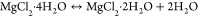
2
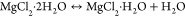
3
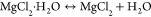
4

**Figure 4:**
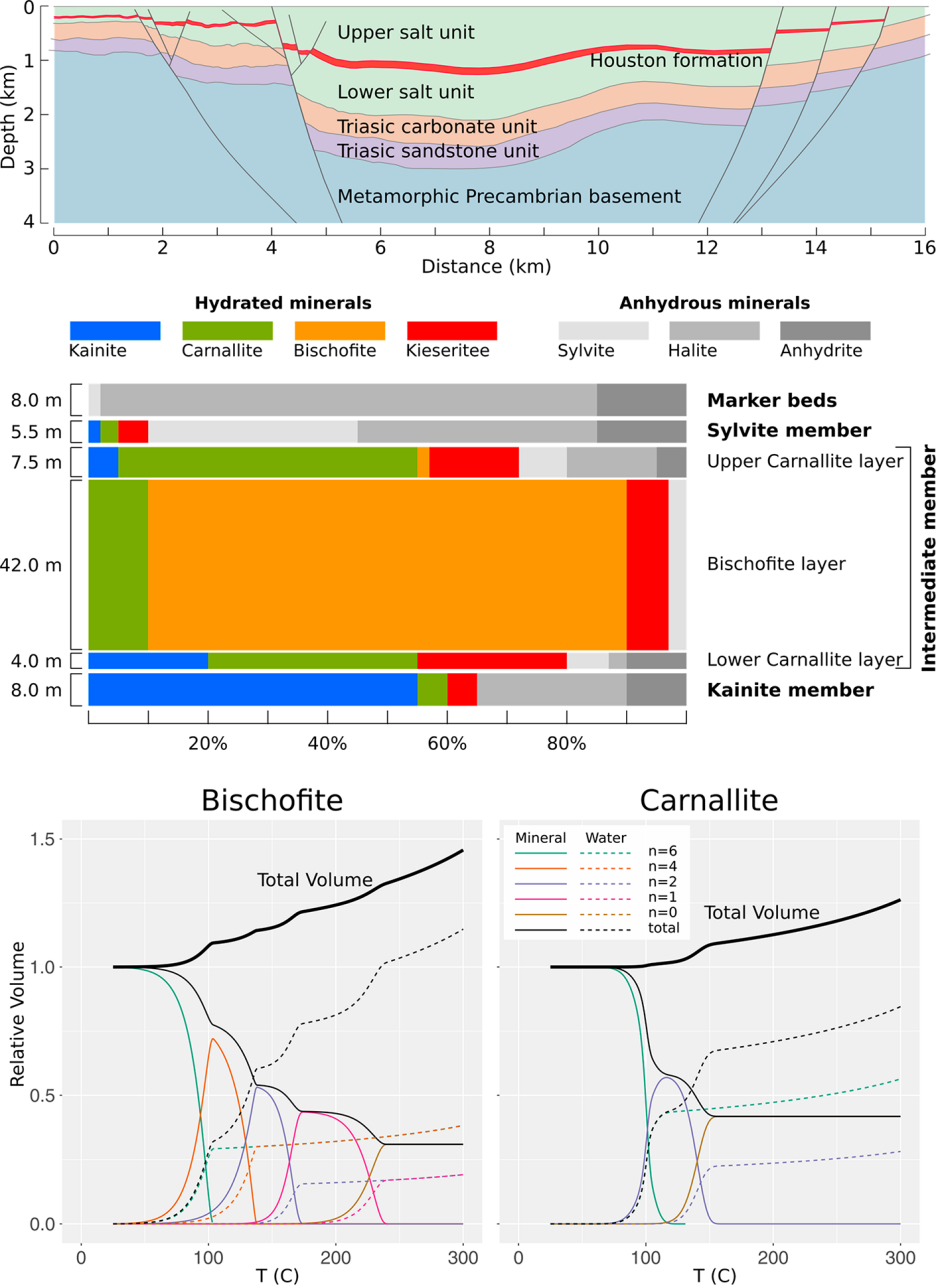
Stratigraphic section (top), composition (middle), and thermal decomposition (bottom) of the Houston Formation within the Danakil evaporitic sequence. Schematic section (E–W) of the Danakil basin stratigraphic sequence near Dallol (top) based on available seismic profiles.^2^ The Upper and Lower salt units, the Houston Formation between them, and the underlying lithologies are shown. The mineralogical composition of the Houston Formation is shown in the middle panel. The vertical size of the bars is proportional to the thickness of the different members and layers (labeled to the right). The *x*-axis represents the relative fraction of each of the different mineral phases. Hydrated mineral boxes are colored, while anhydrous minerals are gray. The area of the boxes is proportional to the amount of each mineral at each layer and in the whole formation. Note that the proportion of the hydrated minerals is very high, especially that of bischofite (45%). The two plots at the bottom show the evolution of the mineral and water relative volume, *V*(*T*)/*V* (*T* = 25 °C), during the thermal dehydration of bischofite and carnallite. The volume of mineral phases is shown as thin solid lines, black for the total volume of solids and colored for each *n*-hydrate. Water volume is shown as dashed lines with the color of the corresponding dehydration reaction. The total volume (mineral+water) is shown as a thick solid line. Hydrolysis reactions are not shown. The total volume upon dehydration of bischofite increases to around 125% of the initial volume at 200 °C, while that of carnallite increases to around 114% of the initial volume at the same temperature. Slow, continuous rise of the water volume clearly seen after the last dehydration step is due to the water density reduction with increasing temperature.

This decomposition produces an increase of volume because the total volume of the solid, dehydrated phase and that of the released water is larger than the initial volume of the equimolar amount of the hydrated phase. This volume increase has been computed by using a function obtained by fitting the recent results from slow thermal gravimetry experiments (TGA) during these transformations^28^ (see the Supporting Information for a detailed description of these procedures and the data used to compute phase density from crystallographic data). Figure 4 (bottom) shows the computed evolution of volume as a function of temperature for the initial, fully hydrated phases, the last, anhydrous phase, and the intermediate phases. The volume of the released water, at each temperature, is also, shown. Dehydration of bischofite produces a volume increase of up to 25% at 200 °C.

The thermal decomposition of bischofite can also proceed, over 150 °C, by hydrolysis. At this temperature, only the dihydrate and the monohydrate produce relevant HCl yields
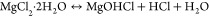
5
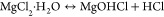
6

The volume balance during carnallite (13% of the Houston Formation) thermal decomposition was similarly quantified from the data provided by several thermoanalytical investigations,^29^ which propose a two-stage decomposition scheme:^40,41^
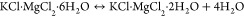
7
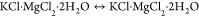
8that allows the computation of solid/liquid volume balances during dehydration. The calculated volume increase during carnallite dehydration reaches 14% at 200 °C.

The thermal decomposition of kainite, the third more abundant mineral, is much more complex than that of bischofite and carnallite. The overall process corresponds to the transformation of four moles of kainite to one of langbeinite, one of kieserite, two of sylvite, and ten of water:^42^
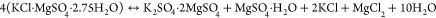
9

The individual steps are complex and can involve reactions with carnallite (2 kainite + carnallite = 2 kieserite +3 sylvite + solution) or kieserite (2 kainite + kieserite = langbeinite + solution). The complexity of this dehydration sequence and the limited crystal-chemical data for some of the phases involved do not allow for the calculation of volume balance, so kainite and kieserite (7.5% of the Houston Formation each) are excluded from the calculations of water balance. The exclusion of kieserite is not expected to have any impact on our results because the available data indicate that their thermal decomposition happens in a single step at a temperature around 325 °C,^43^ i.e., higher than that computed in our thermal model. On the other hand, kainite is expected to partially dehydrate at temperatures around 200 °C, but the quantification of the water released was not possible with the currently available data. Neglecting kainite dehydration could underestimate around 1–5% of the water volume produced by dehydration at the Houston Formation minerals, so the total water volumes in our calculations represent a lower limit for the liquid volumes produced by the thermal destabilization. Even this lower limit for water volumes produced by thermal destabilization is large enough to drive the local doming leading to Mount Dallol and its later evolution.

## 4 Discussion

Explaining the origin Mount Dallol requires a set of processes, consistent with the geological context and the field observations, that are able to produce a local overpressure under Dallol. The existence of a magmatic intrusion in the salt package has been proposed as the driving force for the formation of Mount Dallol and its volcanic-like structures.^15,16,32^ Magmatic/evaporite interactions are not thoroughly investigated due to the scarceness of study localities.^44^ The main conclusions obtained from these scarce sources are that (a) direct magma/salt interactions create distinct but narrow (centimeters to meters) fluid-release envelopes by halite recrystallization and fluid migration from the inclusions,^45^ (b) beds rich in hydrated minerals get destabilized at temperatures far below the halite melting point and, (c) the behavior of levels containing a high fraction of hydrated salts is different due to dehydration; for instance, thermal water release from a 2 m thick bed of carnallite has been reported to create large mobilization of materials and thixotropic or subsurface “peperite” textures.^46^ In addition, previous literature^2,27^ reporting seismic profiles and drill core studies of the Danakil evaporitic sequence does not show the presence of important shallow (i.e., within the 2 km thick evaporitic sequence) magmatic emplacements under the Dallol area, making impossible extensive, direct contact between basalt and salt. On top of that, our thorough characterization of the lithological units of Mount Dallol and the nearby locations show a consistent absence of silicate minerals (other than minor sedimentary clays) of effusive or explosive origin, even within breccias. With these premises, explaining the formation of Mount Dallol requires a model starting with the generation of a nonmagmatic, pressurized fluid at temperatures under 300 °C that drives the up-doming of Mount Dallol, and any later effusive and explosive events.

**Figure 5:**
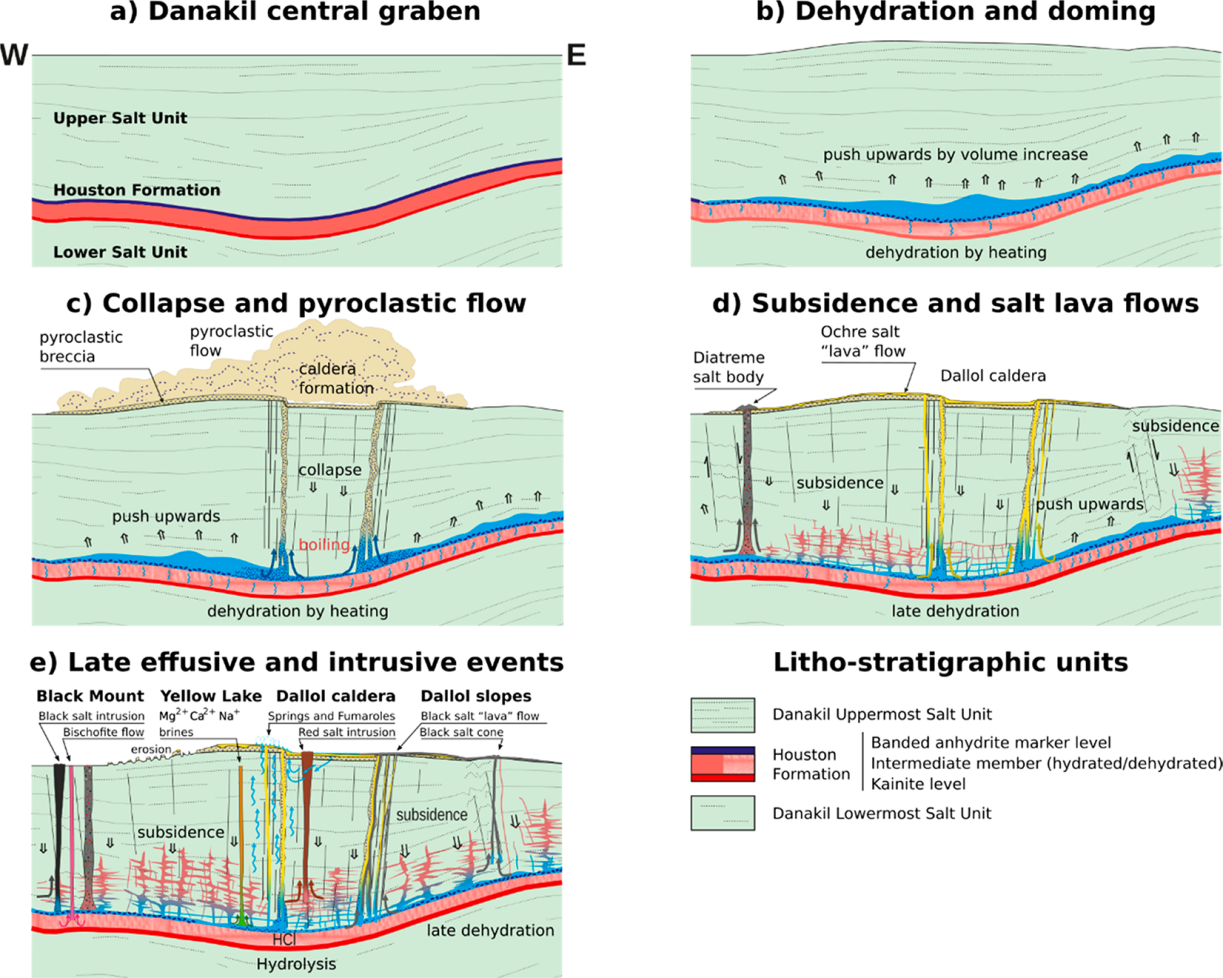
Sketch of the geological evolution of Mount Dallol. (a) Danakil evaporitic sequence at the Dallol central graben before the magmatic intrusion. (b) Dehydration reactions begin to occur at *T* > 100 °C at the Houston Formation 30,000 years after the magmatic intrusion. The increase in the total volume of the partially dehydrated salts and the water produced pushes upward the upper salt unit. (c) Circular caldera is formed due to the collapse of the dome. The depressurization of the brine chamber releases a “clastic breccia” (anhydrite, shales, and clays) that covers most of the dome. (d) Fluids in the brine chamber outflow through the fractures as an ochre salt lava around the caldera, at the dome slopes. Subsidence continues; new faults and deformed regions lead to further depressurization and local emplacement of explosive bodies (diatreme structures of salt breccias) around the dome. (e) Salt effusive events still occur. The less viscous materials flow through the Eastern part, forming the Black salt flow. The more viscous materials form intrusive bodies (i.e., Chocolate facies and Black Mound). Leaking gases produce small local explosive events through deep chimneys. Rehydrated bischofite suspensions outflow nearby the Black Lake. Salt dehydration in the Houston Formation is almost completed. The last decomposition stages occur by hydrolysis reactions, releasing HCl that lead to the settlement of the hyper-acidic hydrothermal system at the Dallol caldera.

Water is contained within the salts of the Danakil sequence in different amounts and forms. Halite contains abundant intergranular fluid inclusions and solution-filled pores, typically around 1% in weight.^47^ Thermally driven recrystallization of halite close to magma/salt contacts has been reported.^44^ This process produces textural changes, from chevron structures to clear crystals, and fluid-inclusion migration. The water released by the large amount of halite in the evaporitic sequence would be more than enough to form the Dallol dome, but, even when assuming a direct magma/salt contact, thermal recrystallization requires high temperatures that can only be reached close to a salt-magma contact; these contact aureoles with extensive halite recrystallization are known to only extend a few meters from the salt/magma contact.^44^ The alternative source of water that we propose in this work is the up to 70 m thick Houston Formation, containing a huge amount of highly hydrated minerals (Figure 4). Our results show that a basaltic intrusion, at a depth consistent with the existing seismic profiles, can raise the temperature of the Houston Formation to a level that would drive the dehydration of the major hydrated minerals in this formation (100–220 °C) and that the thermal decomposition of these minerals would lead to a volume increase of, at least, 25%. The Dallol dome (approximately a spherical cap 3.5 km in diameter and 60 m in height) can be formed by the partial dehydration (at 200 °C) of the Houston Formation under a circle of radius 3.4 km centered in Mount Dallol. We propose that the generation of this volume of water upon dehydration is responsible for the elevation of the Dallol dome and the subsequent formation of a salt volcano when the resulting brine reached the surface. The corresponding series of events is illustrated in Figure 5.

Dehydration water would most likely be accumulated in a “brine chamber” located ca. 1000 m deep, just above the Houston Formation, at a temperature from 150 to 220 °C. The dehydration of minerals will partially dissolve the surrounding salt volumes, generating a saturated brine and a smaller volume of anhydrous minerals. Both the solid volume reduction and the dissolution lead to the increase in porosity required for the settlement of the brine chamber. As dehydration starts in the deeper parts of the Houston Formation and progresses sideways with further temperature increases, a lateral gradient in porosity also develops, which drives the lateral migration of brines toward the central brine chamber. This chamber contains a saturated, viscous, brine slurry under a dome structure produced by the volume increase (Figure 5b). The pressure in this chamber can be calculated as the lithostatic pressure produced by 1000 m of halite: *P* = *ρgh* = 2160 × 9.81 × 1000 = 21.19 MPa. Under these conditions, water is liquid^48^ but it can transform to vapor if the temperature reaches at least 370.6 °C. This value corresponds to pure water; the actual temperature, dependent on salinity, will be higher, which is very unlikely within the limits of our thermal model. Alternatively, boiling is also possible if the pressure is reduced below 0.48 MPa.^32^

The doming processes lead to an unstable situation in which buoyancy forces develop. The raised dome stands on a mechanically unstable salt volume, subject to extensional faulting and containing large amounts of brine-filled spaces. Unconfined extrusion is the most suitable mechanism to explain diapiric, slow emplacement in mud volcanism and vertically constrained intrusions in the case of mèlanges.^49^ In the case of Dallol, since the viscosity of the brines is expected to be smaller than that of mud, the unconfined extrusion mechanism could be the most reasonable except in the case of the highly viscous slurries of the Chocolate facies. Fractures generated by either doming deformation, hydrofracturing due to pressure build-up, or extensional faults would allow for the upward migration of the brines in the chamber and their eventual depressurization. The combined effect of fracturing and brine migration produced the collapse of the central part of the dome and the subsequent formation of a collapse caldera limited by circular faults or radial fractures currently observed in Dallol (Figure 5c). During this collapse, when the pressure at the brine chamber and cavities along fractures falls below 0.48 MPa, the brines start boiling, and the vapor rises through the faults, sweeping along fragments of the most insoluble salts. At the surface, this mix of vapor and insoluble salts forms a clastic flow evidenced by the frequent breccias observed at the dome, which have a characteristic internal structure of typical pyroclastic deposits^37^ (Figure 5c). Their composition (blocks of the more insoluble minerals, mostly anhydrite, clays, lutites, etc.) indicates that they were produced by continuous, vapor-pushed emissions during the formation of the caldera, driven by the further volume increase produced by vaporization of the raising brines, rather than by large explosive events. Breccias previously, reported in mud volcanoes are the result of fault breccias and hydrofractured blocks, being transported to the surface by the rising mud.^26^ These breccias contain a mud matrix (in variable amounts), but the breccia in Dallol does not contain appreciable amounts of halite or other soluble salts in the matrix, so their origin should be explained in terms of pyroclastic-like processes involving the dissolution of salts during the upward transport of rock fragments. After the formation of the Dallol caldera, the materials into the brine chamber flowed to the surface through the annular fractures, pushed by the subsidence of the sinking segments of the central dome (Figure 5d). This effusive process carried to the surface saturated, viscous brines bearing halite crystals and fine insoluble materials with mechanical properties close to that of lava flows. Field observations show that these flows occurred after the breccia deposits were partially eroded (Figure 2c and Figure S3a). Three effusive pulses produced the ochre and the black salt flows (Figure S3c), and a subintrusive body inside the caldera, the brown-reddish salt (“Chocolate facies”). The different colors of these flows indicate differences in the content of fine insoluble materials and of iron and its oxidation state, with the corresponding increase in viscosity and likely a decrease in the temperature of the brines. The so-called “Chocolate facies” in the central part of the caldera are the result of the emplacement of a highly viscous brine-slurry from the chamber.

Meanwhile, at the Houston Formation, above 150 °C, hydrolysis reactions (mainly those in eqs 5 and 6) became much more active than the dehydration ones, releasing large amounts of HCl. According to our model, this should be occurring nowadays. Mobilization of this HCl, along with volatiles contributed by the underlying magmatic materials (CO_2_, SH_2_, SO_2_), is the origin of —or a substantial contribution to— the currently active hyper-acidic hydrothermal system set on top of the Dallol salt volcano. Our model also accounts for other noticeable features in the area, namely, the Black Lake, bischofite flows close to the Black Mountain, the Yellow Lake, and other almost dismantled domes, such as the Red Mound, located to the east of Dallol (Figure 2a).

This sequence of events is driven by the generation of brines by thermal destabilization of the hydrated salts in the Houston Formation under Dallol. Our calculations are approximate but accurate enough to fully support our conclusions and show that these dehydration reactions are able to produce the doming and the effusive processes observed in Dallol. The main simplifications used in our calculations are (a) the use of experimental thermoanalytical data instead of theoretical phase diagrams, (b) neglecting the formation of saturated brines during dehydration, and (c) not considering the pressure as a factor shifting the transformation reactions. Unfortunately, the literature on phase diagrams is scarce except for bischofite and carnallite, which are used as energy storage materials, and even in these cases, phase diagrams are restricted to atmospheric pressure. The transition temperatures from recent phase diagrams agree with those used in our calculations. Recent phase diagrams^50^ show that the hexahydrate–tetrahydrate transition happens at 389.85 K and that of tetrahydrate–dihydrate at 454.65 K. In our model, derived from thermoanalytical data,^28^ these temperatures are 380 and 445 K, respectively (Figure S7). Those differences are smaller due to the uncertainties in the depth and size of the magmatic intrusion producing the heating. In addition, transition temperatures are known to change with the impurity content,^28,51^ which is highly relevant to the problem at hand; for example, the experimental data used in our calculations were taken from a publication reporting two different TGA curves, one for natural bischofite and one for synthetic MgCl_2_·6H_2_O, with different transition temperatures and transformation fractions.^28^ Any of those, or even new data, can be applied to our calculation methodology, while only the pure phase transitions can be computed using phase diagrams.

The decomposition of MgCl_2_ hydrates happens by incongruent melting, involving the simultaneous liberation of water and dissolution of mineral into a saturated brine. To assess the impact of neglecting brine formation in our model, we have checked the volume balance during transformation of the tetrahydrate to the dihydrate, which spans most of the temperature range considered in our work. Recently published phase diagrams^50^ show that the MgCl_2_·6H_2_O + MgCl_2_·4H_2_O invariant point is at 389.85 K and 46.2 wt % MgCl_2_, while the MgCl_2_·4H_2_O + MgCl_2_·2H_2_O invariant point is at 454.65 K and 55.8 wt %. MgCl_2_. Assuming that this transformation happens by incongruent melting, we can compute the changes in the solid and the solution during heating from one to the other, which correspond to the transformation of 1.01 mol of MgCl_2_·4H_2_O per kilogram of water; this solid gets dissolved in the water released during the transformation. Between both invariant points, 168.94 g of the tetrahydrate is dissolved. Using a corresponding density of 1.638 g/cm^3^ (Table S1), this corresponds to 103.14 cm^3^. The solution produced between both points contains 72.80 g of additional water and, according to the published data on the *P*/*T*-dependent density of MgCl_2_ solutions,^52^ the density of this solution (5.00 mol·kg^–1^, *T* = 472.96 K and *P* = 19.97 MPa) is 1222.30 kg/m^3^, so the respective volume is incremented in 138.26 cm^3^. The net balance in volume is therefore an increase of 138.26–103.14 = 35.12 cm^3^, around 34%, in good agreement with our model calculations.

The last simplification to be checked in our model is neglecting the effect of pressure into the phase transition temperatures, the solubility of the phases, and the density of brines. There is little information on this effect for most of the phases considered, but the melting temperature of bischofite is pressure dependent, practically linear up to 500 MPa and increases 1 °C per 10 MPa,^53^ which can be safely neglected in our case, at an initial pressure of 20 MPa, which is later reduced upon cracking at the beginning of effusive processes. The effect of pressure on the solubility of the salts can be estimated from the corresponding equilibrium constant at atmospheric pressure and the molar volume increment (around 35 cm^3^ per mole as computed above).^54^ For the MgCl_2_·4H_2_O dissolution reaction, log *K* changes from 3.455 at 200 °C and *P* = 1 atm to 3.378 at 200 °C and *P* = 20 MPa. The dissolution of the hydrated salt is inhibited by pressure, but the change is small enough to be safely neglected.

Except for the origin and composition of the mobilized fluids, the sequence of events leading to the formation of Mount Dallol is very close to the one producing an ordinary volcano: creation of a pressurized fluid chamber, local doming, release of clastic materials, caldera formation, and effusion of the contents of the chamber. The hyper acidic, salt-saturated hydrothermal system that awards Dallol its current geochemical singularity and beauty is the last step of this sequence, namely, the circulation of hydrothermal solutions through the crack produced by the doming and the caldera collapse and their acidification by thermally driven hydrolysis reactions. In the future, this salt-volcano and the accompanying hyper-extreme hydrothermal system will disappear as rifting advances, most probably replaced by a basaltic shield volcano similar to those near Erta Ale Range. The salt-volcano of Dallol is a transient evidence of how mineralochemical processes leading to the generation of pressurized fluids that eventually reach the surface can produce volcanic-like features and edifices. The same type of processes may contribute to other types of atypical volcanoes on Earth and elsewhere, like the “mound-like landforms” in Mars described as mud volcanism breccia deposits on top high albedo deposits disrupted in meter-sized polygons,^55^ which closely resemble Mount Dallol: a volcano-like mound on top of the polygonal white salt crusts of the Danakil salt plain. Revisiting these Martian features on the basis of the model proposed here might also contribute to our knowledge of planetary geology.

## Supporting Information Available

Figures of Google-Earth satellite image of NW branch of Afar triangle where is located the Danakil Depression, pictures of Pyroclastic structures on top of the main Dallol dome, massive salt structures on top of the main Dallol dome, and breccias near the main Dallol dome, TGA curves, output of the successive stages during TGA data processing, moles of each of the mineral phases present during the thermal decomposition of one mole of bischofite, moles of water produced by the dehydration of the hexa-, tetra-, di-, and monono-hydrate, volume of the different solids and fluids involved in the thermal decomposition of bischofite, volume of the different solids and fluids involved in the thermal decomposition of carnallite, density of water and a solution resulting from the dissolution of all the HCl released by hydrolysis reactions, concentration resulting from dissolving the HCl released by the hydrolysis of the mono-hydrate into the water, thermal diffusivity as a function of temperature, representation of the array of integers encoding the lithology during calculations, representation of the k-array, and representation of the discrete temperature array during the last iteration of the calculation, discussions of thermal decomposition of the Houston Formation, and thermal evolution of the Dallol area, and tables of crystallographic data used for density calculations of phases involved in the thermal decomposition of bischofite, crystallographic data used for density calculations of phases involved in the thermal decomposition of carnallite, and parameters of the thermal model (PDF)
Movie of time evolution of the heating event (MP4)
