# Supplementary material for: Mineralochemical Mechanism for the Formation of Salt Volcanoes: The Case of Mount Dallol (Afar Triangle, Ethiopia)
Source: ACS Earth Space Chem. 2022 Oct 19;6(12):2767–78. doi: 10.1021/acsearthspacechem.2c00075 (PMC9761782; doi:10.1021/acsearthspacechem.2c00075)
Supplement: Supplementary file 1 — sp2c00075_si_001.pdf [file sp2c00075_si_001.pdf]

A MINERALOCHEMICAL MECHANISM FOR THE FORMATION OF SALT VOLCANOES: THE CASE OF MOUNT DALLOL (AFAR TRIANGLE, ETHIOPIA)

Fermín Otálora, Fernando Palero, Evgenia-Maria Papaslioti & Juan Manuel García-Ruiz*

Laboratorio de Estudios Cristalográficos. Instituto Andaluz de Ciencias de la Tierra. CSIC-Universidad de Granada. Granada, Spain, 18100.

*jmgruiz@ugr.es

This document contains additional information and figures on the data and methods used in the article "A MINERALOCHEMICAL MECHANISM FOR THE FORMATION OF SALT VOLCANOES: THE CASE OF MOUNT DALLOL (AFAR TRIANGLE, ETHIOPIA)" by F. Otálora, F. Palero, E.M. Papaslioti & J.M. García-Ruiz. Contents:

1. Supplementary figures on the different structures and lithologies of Dallol.
2. Thermal decomposition of the Houston Formation: Methodology used for the computation of the volume increases due to dehydration. Tables of crystallographic information used for the calculation of mineral densities and volume balance.
3. Thermal evolution of the Dallol area: Description of the two-dimensional finite differences. Calculation of the transient and steady state thermal gradients below Dallol after the magmatic emplacement. Lithology and temperature-dependent parameters used for the calculations. Code listings and description of the software used.

1. Lithology and structures of Dallol

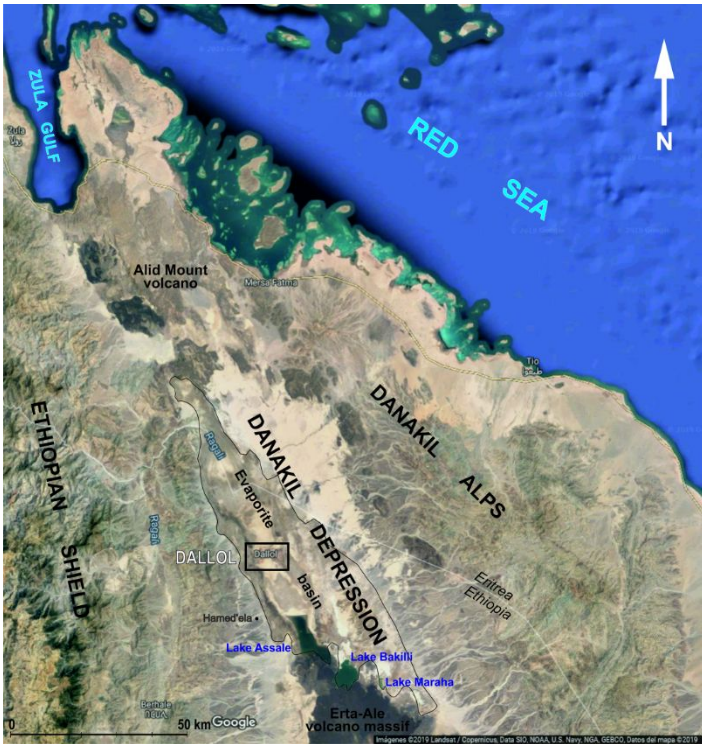

Figure S1: Google-Earth satellite image of NW branch of Afar triangle where is located the Danakil Depression. An important part of the depression is occupied by the salt pan, forming a huge and thick evaporite basin. The basin lies between the high-lands of Danakil Alps at East and the Ethiopian Shield at West.

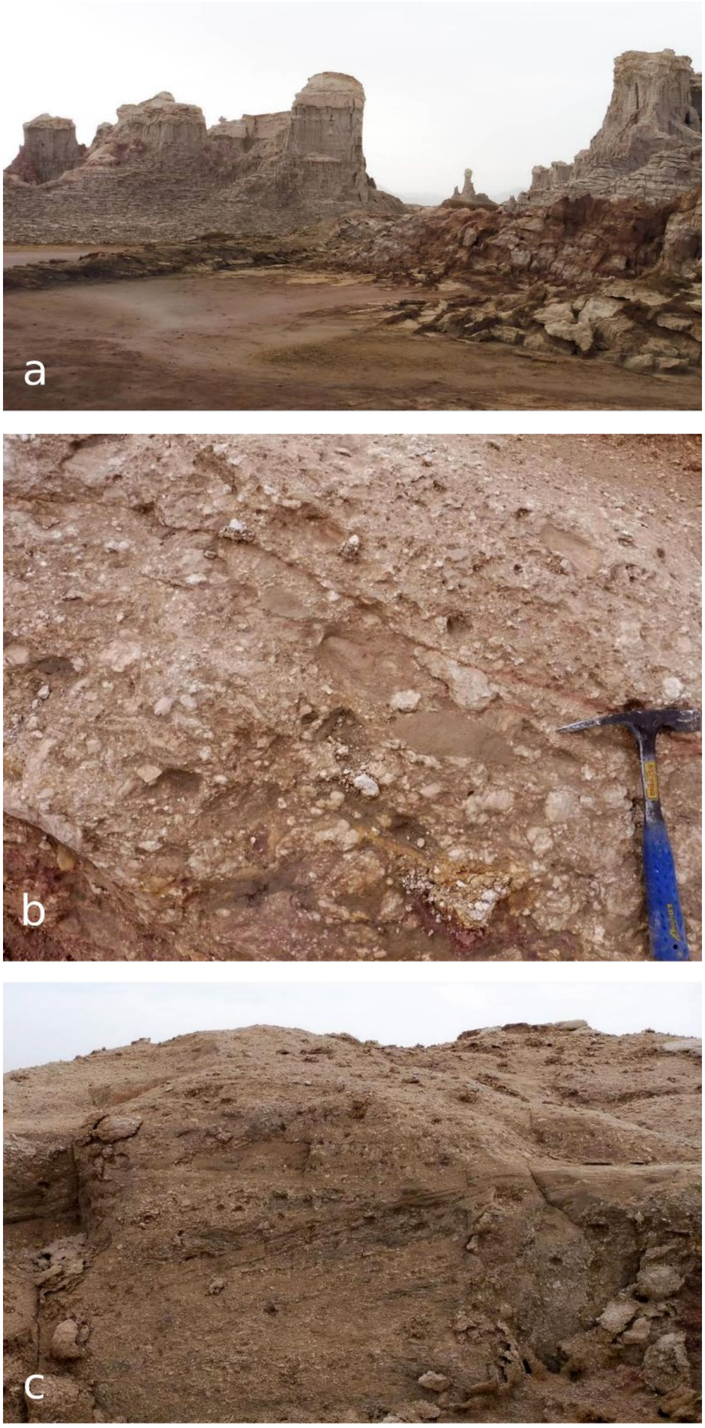

Figure S2: Pyroclastic structures on top of the main Dallol dome. a, Ruin-form landscape in the western part of the Dallol Dome, developed by dissolution of a halite multilayer of the Danakil evaporite sequence. Above of the salt sequence there is a breccia layer formed by fragments of anhydrite, shales, clays and lutites that act as a resistant level in the "demoiselle coiffée" structures, protecting the relics of the saline material from the rainfalls. **b,** Chaotic breccia with fragments of anhydrite, shales and clays surrounded by a clay matrix. It corresponds to the basal section of the breccia package that appears on the halite alternation of the Upper Salt Formation. **c,** Breccia with some layered arrangement with parallel lamination and cross stratification of the roof section of the packet of anhydrite breccias, shales and loamy clays that appears over the halite alternation of the Upper Salt Formation.

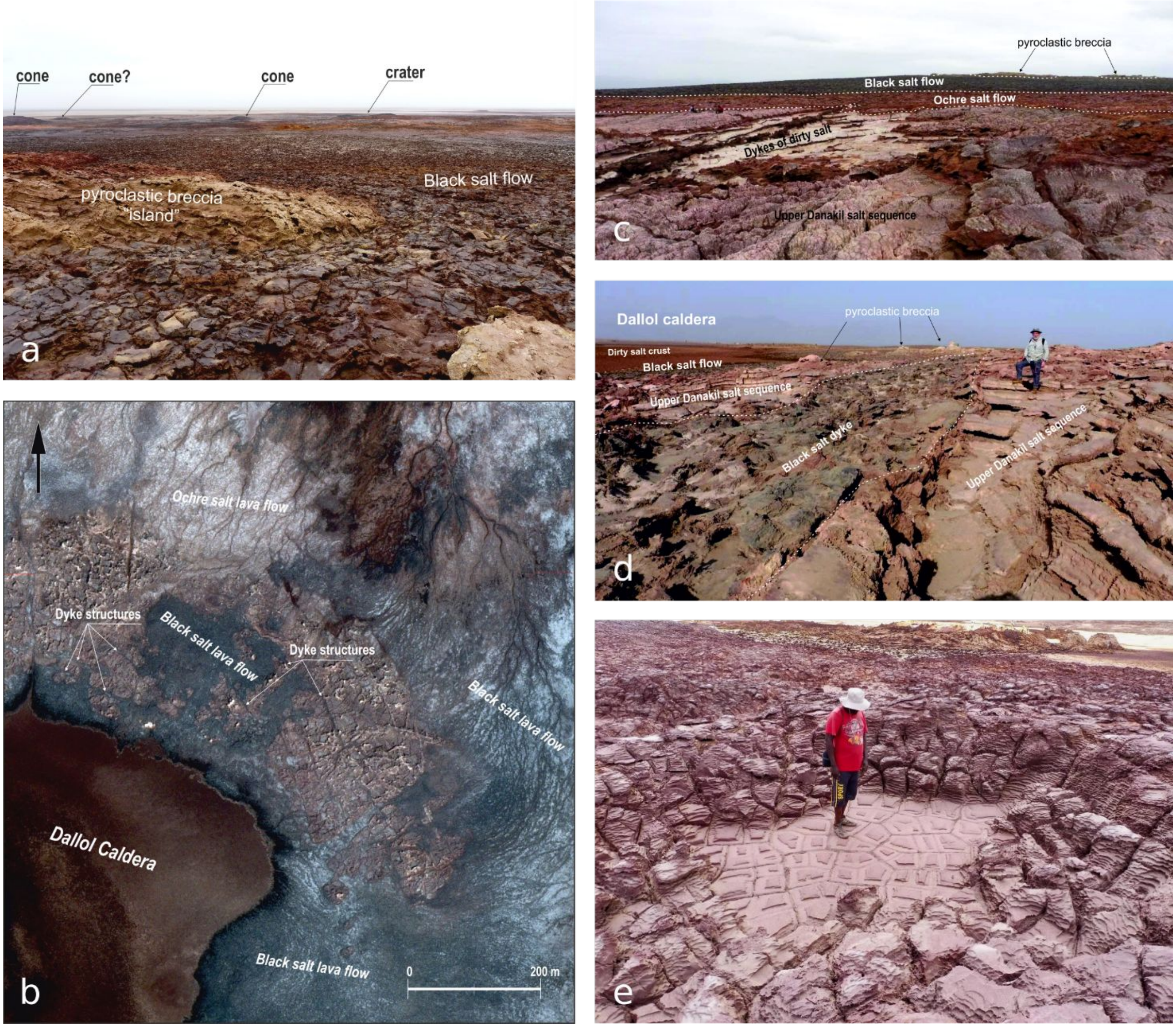

Figure S3: Massive salt structures on top of the main Dallol dome. **a,** Black salt flow along the edge of a pyroclastic breccia raised as an island, and some effusive structures in the East side of Mount Dallol. **b,** Fragment of the DEIMOS image of the NE corner of the Dallol caldera, showing the lava flow of the black salt and the dikes of the same material embedded in the alternation of the Upper Salt Formation. **c,** Massive dark gray salt stream (top) superimposed on the massive brown salt stream (middle strip), in turn covering the alternating salt layers of the Danakil evaporite sequence (bottom of photo), in the Southeastern slope of the Dome of Dallol. **d,** Massive dark gray salt intruding on alternating salt layers of the Danakil evaporite sequence. On the left is the caldera of the Dome of Dallol with the reddish brown salt crust caused by drying out of the lagoon that forms during the rainy season. Note the change in dip of the alternating salt layers on either side of the gray salt dam, towards the center of the caldera on the left and out of the dome on the right. **e,** Structuring as a tiled soil or chocolate bar in the massive body of the reddish salt inside the Dallol Caldera. These forms are produced by the dissolution in favor of the joint with geometric pattern of this massive and homogeneous saline material, called ''Chocolate Facies''.

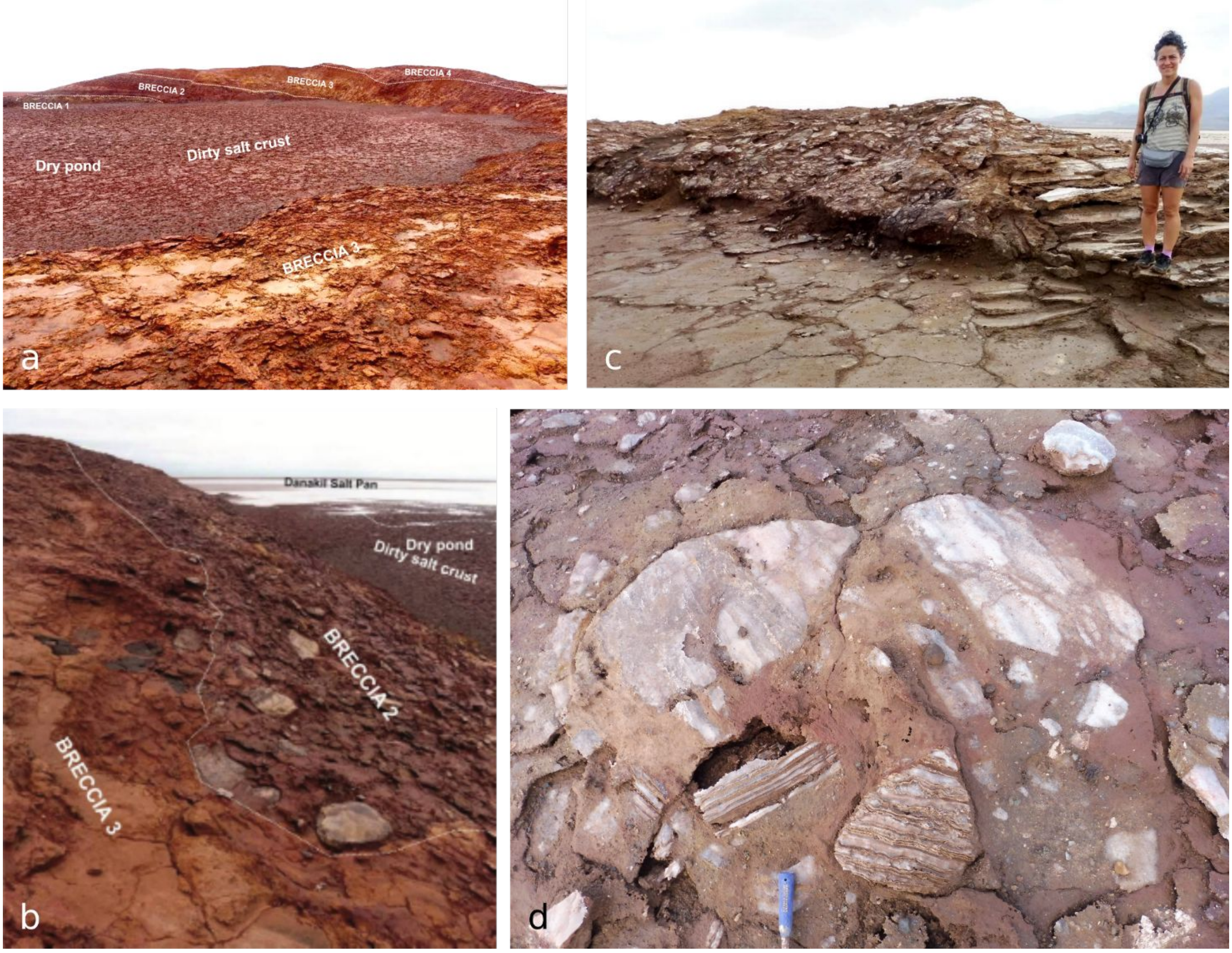

Figure S4: Breccias near the main Dallol dome. a, The colour of the massive salt as the matrix in the breccia layers highlights the different strata. In the centre of the structure, rainfalls during the wet season give rise to a pond that gathers the insoluble material (mainly clays), which forms a dirty salt crust with reddish colour after evaporation. **b,** Outcrops of explosive breccias in the rim of the Horseshoe Mount. In the photo we can see two strata of breccias formed by blocks of different types of salt, giving a different colour to the two layers. **c,** Megabreccia body of white salt fragments enveloped by a dirty brownish and reddish salt matrix. On the right we see the net contact with the alternating layers of Danakil halite appearing on the SW edge of the Dallol Dome. **d,** Large (up to 1m) blocks of different types of salt forming a macro-breccia, infilling the diatremic body near Black Mount. Blocks of salt from the sedimentary sequence prevail in this breccia.

2. Thermal decomposition of the Houston Formation

2.1. Methodology for computing volume increases by dehydration

Many previous works (mainly on the engineering of industrial Mg production from bischofite) on the thermal decomposition of hydrated magnesium chloride phases have been published; e.g. see references in Table 1[1],where are compiled studies on the dehydration and hydrolysis reactions. The TGA data from that work (see Fig. 7 of ref[1]) have been used for the following calculations. In the present study, two different samples were used: natural bischofite from Antofagasta Region, Chile, obtained from the brines concentration process and

Merck-branded synthetic (> 99% purity) bischofite. The dehydration curves for both samples are shown in **Fig. S5**. The main differences between the two samples include the weight loss, that is from 5% to 10% less in the natural sample, and the first dehydration step, that goes to the tetrahydtate in the syntetic sample and to the pentahydrate in the natural one. Both differences can be attributed to the presence of impurities present in the natural sample (NaCl, $Li_2SO_4 \cdot H_2O$, KCl and $KCl \cdot MgCl_2 \cdot 6H_2O$)[1], leading to a reduced weight loss due to the presence of anhydrous minerals, and to a complex thermal decomposition due to the presence of other hydrated minerals. Since we are interested in the behavior of pure bischofite, while chemical, thermodynamical and structural (density) data are not available for the hypotetical pentahydrate, we used the synthetic sample for presenting the following results.

These TGA data were processed for bischofite, and for other hydrated phases below, by:

1. **Digitizing** the plot data. Using the software xyscan[2] from the Yale University, a set of representative (T, weight loss) points (24 to 40) on the curve was recorded.
2. **Interpolating** the data. From these points, a data set was spline-interpolated using the software R[3] into a equispaced data set for T=25,26,27, …499,500.
3. **Differentiation**. The first derivative of the interpolated dataset was computed using R so that each phase transition shows as a peak spanning the corresponding temperature range and having a maximum at the temperature of the maximum transformation rate.
4. **Fitting**. These derivatives were fitted (using R) to the sum of n Exponential-Gaussian Hybrid distributions to account for peak asymmetry[4], n being the number of decomposition steps of the given phase.
5. **Integration**. The resulting peaks were integrated separately to obtain smooth, noise-free, zero-background, cumulative weight loss for each dehydration steps. These functions, after normalization to remove experimental/theoretical differences (always smaller than 10%), are the final product of experimental data processing. The distribution of all mineral phases, the yield of the decomposition reaction, and the volume balances were computed using those functions. Weight loss curves in units of % mass, grams and moles were computed at this stage.
6. **Separation** of parallel reactions. For the case of the $MgCl_2$*H_2O decomposition, as discussed above, the parallel equations 4 and 14 were taken into account. The weight loss function obtained in the previous

step was decomposed in two for the respective reactions using the T-dependent $P_{14}^{HCl}/P_{14}^{H2O}$ ratio as explained before.

Figure S5 shows the original TGA data for bischofite as reported in ref[1]. Using these data and following the process described above and illustrated in **Fig. S6**, we obtained the amount of each of the mineral phases and liquids illustrated in **Figs S7** and **S8**.

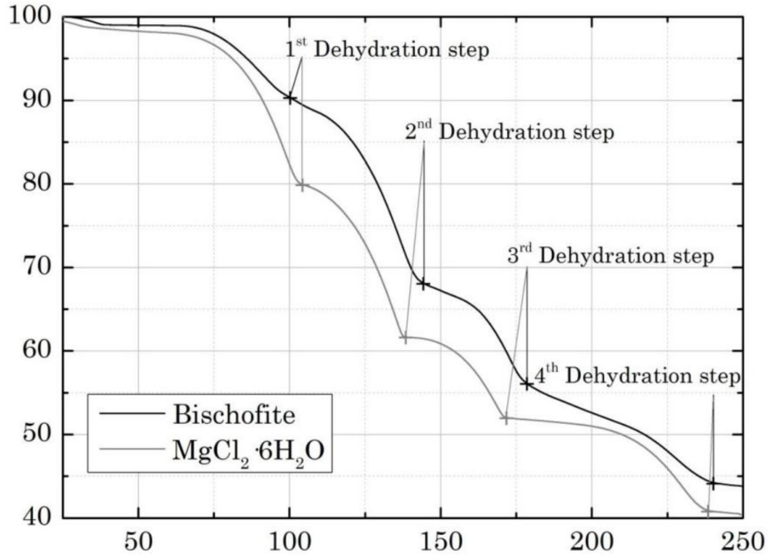

Fig. S5: TGA of bischofite and $MgCl_2{\cdot}6H_2O$ decomposition (heating rate 1 K/min)[1].

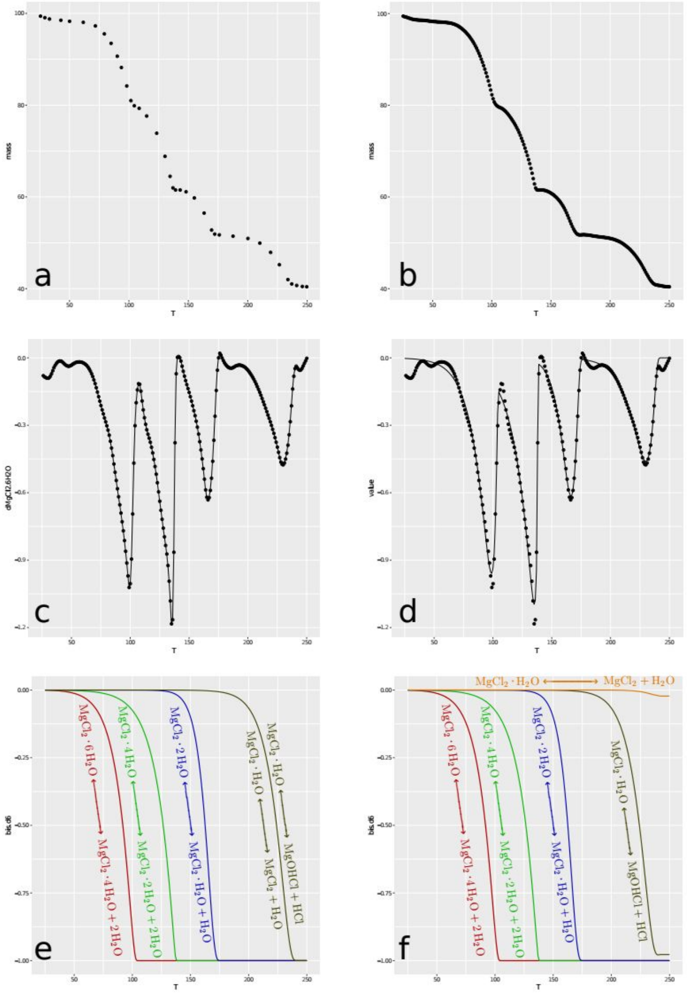

Fig. S6: Output of the successive stages during TGA data processing. a, Starting with the experimental data from ref[1] **(Fig. S7)**, the curve for the synthetic sample was digitized as a series of mass loss vs. temperature . **b,** These data are then interpolated to obtain a curve with data at equally spaced T values . **c,** The derivative of this curve is computed, and **d,** fitted to a sum of four Exponential-Gaussian Hybrid distributions, **e,** These are then integrated to obtain the curves for the molar decomposition at each of the four decomposition stages. **f,** by quantifying the relative rates of each of the monohydrate reactions, the last step is decomposed in two different curves.

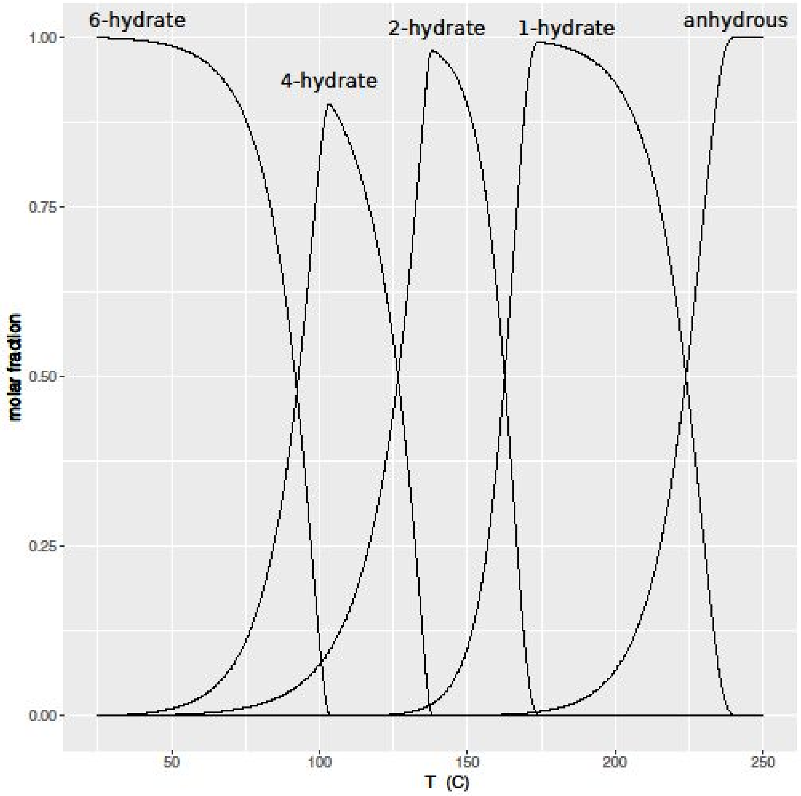

Fig. S7: Moles of each of the mineral phases present during the thermal decomposition of one mole of bischofite.

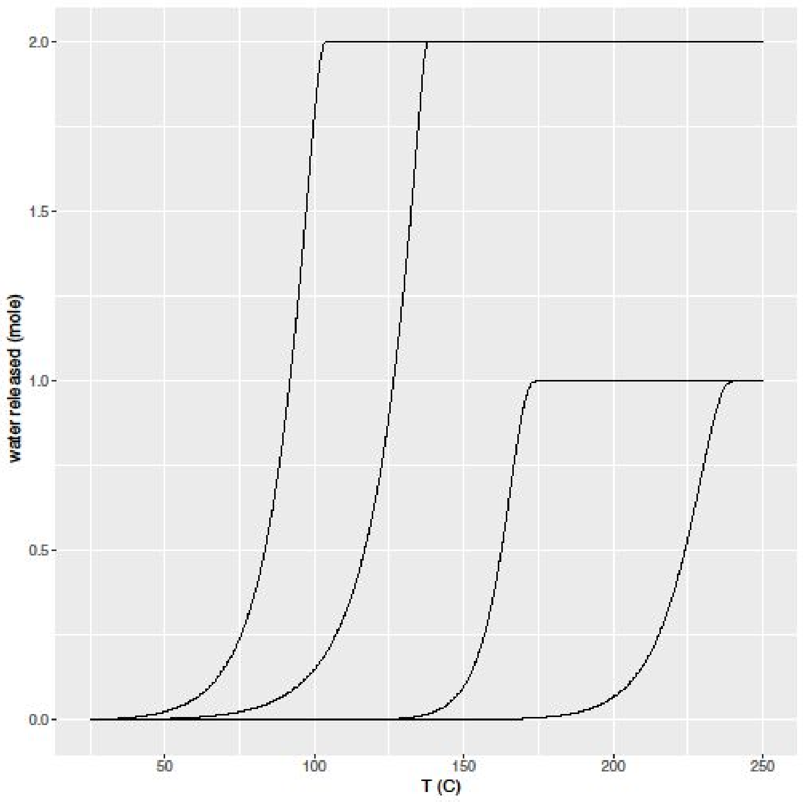

Fig. S8: Moles of water produced by the dehydration of the hexa-, tetra-, di- and monono-hydrate from left to right.

To convert these mole fractions into volume fractions, the density of the different phases must be considered. The density data used in this work were computed from crystallographic data, (multiplicity times the molecular weight divided by the unit cell volume) obtained from the American Mineralogist Crystal Structure Database[5]. Data for the oxychloride are not available from this database, and were obtained from the PAULING FILE Multinaries Edition one[6]. Data for the hexa-hydrate are from ref[7], and for the tetra- di- and mono-hydrates from ref[8]. **Table S1** summarizes the data used.

Table S1: Crystallographic data used for density calculations of phases involved in the thermal decomposition of bischofite.

phase	mol w.	symm	sp. gr.	a	b	c	α	β	γ	volume	mult.	density
MgCl 2 □6 H 2	203.3	mono	C12/m1	9.8607	7.1071	6.0737	90	93.758	90	424.736	8	1.59
MgCl 2 □4 H 2	167.27	orth	Cmcm	4.21616	11.023	7.2951	90	90	90	339.04	2	1.638
MgCl 2 □2 H 2	131.24	mono	C2/m	7.4279	8.5736	3.65065	90	98.58	90	229.89	2	1.896
MgCl 2 □H 2 C	113.23	orth	Pnma	8.9171	3.63421	11.4775	90	90	90	371.95	4	2.022
MgCl 2	95.21	trig	R-3m	3.596	3.596	17.589	90	90	120	196.975	3	2.408
MgOHCl	76.77											2.2

The density of water as a function of pressure and temperature was computed using the IAPWS Formulation 1995[9] implemented in the R package IAPWS95[10]. The P/T dependency of the liquid/gas boundary, the triple

and critical points, defining the state of water in the brine chamber, and the response of these fluids to heating and decompression, were obtained from the same source.

Volume fractions were calculated using data in **Table S1**. **Figure S9** summarizes the evolution of the thermal decomposition of bischofite in terms of volume changes.

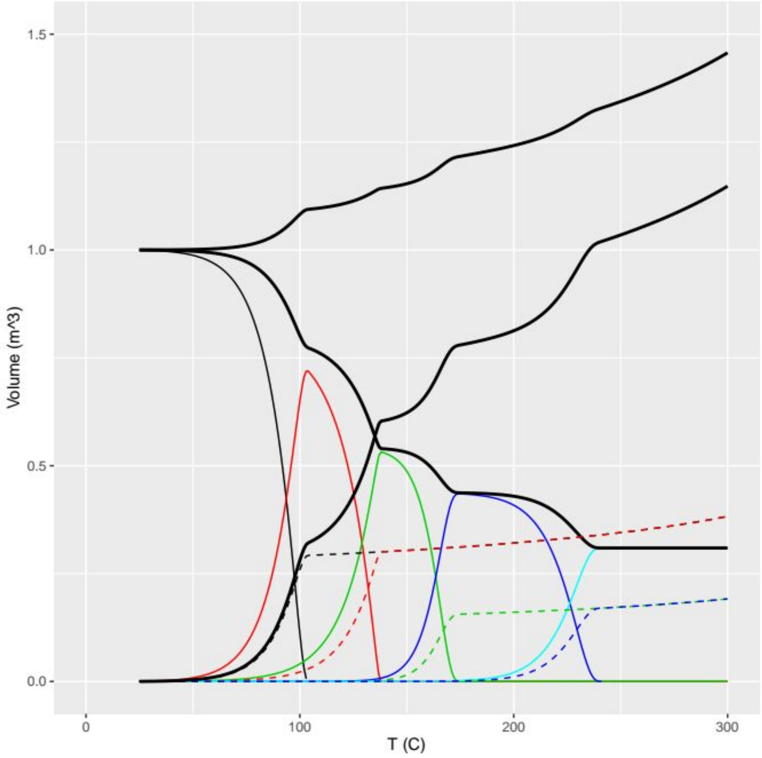

Figure S9: Volume of the different solids and fluids involved in the thermal decomposition of bischofite. The initial point is 1m^3 of pure bischofite. Successive dehydration of this volume of hexa-hydrate (black thin solid-line curve) produced a decreasing volume of the tetra- di- and mono-hydrate (thin solid-line red, green, blue and cyan curves, respectively) and the corresponding volume of water (dashed lines of the same color). The volume of HCl is not plotted because it is produced as gas that get dissolved in the water, increasing non-linearly the water volume. The water volume increased even after the decomposition due to the reduction of water density with increasing temperature. The solid lines show total volumes (for solids, fluids and total).

We have modeled the two-step dehydration of carnallite, according reactions to 17 and 18 and using the same methodology described for bischofite. The only difference is that the peak corresponding to the first dehydration has a wide foot, which does not fit well with a EGH peak. Matching it to the sum of two such curves improves a lot the coordination of the overall decomposition.

In the case of carnallite, volumes computed from this model were based on the crystallographic data shown in **Table S2**. Data for the hexa-hydrate were obtained from the American Mineralogist Crystal Structure Database (database code "amcsd 0001005")[11]. The data for the anhydrous chloride were obtained from the JCPDS no. 01-074-2233 reccord[12]. No unit cell information is included in this record, just an unindexed list of powder diffraction peaks[13]. From this list we have computed a unit cell and indexing list using the dicvol[14] software. This unit cell is the one listed in **Table S2** and used in the calculations.

Using these data, the volume balance upon thermal decomposition of carnallite has been computed using the same methodology as in bischofite. The result of this model is shown in **Fig. S10**.

Table S2: Crystallographic data used for density calculations of phases involved in the thermal decomposition of carnallite.

phase	mol w.	symm	sp. gr.	a	b	c	α	β	γ	volume	mult.	density
KCl □MgCl 2 □	277.85	orth	Pnna	16.119	22.472	9.551	90	90	90	3459.62	12	1.6
KCl □MgCl 2 □	205.79	mono	Pbnm	11.134	3.234	9.338	90	95.822	90	334.46	2	2.04
KCl □MgCl 2	169.76	orth		6.971	9.922	6.954	90	90	90	480.98	4	2.34

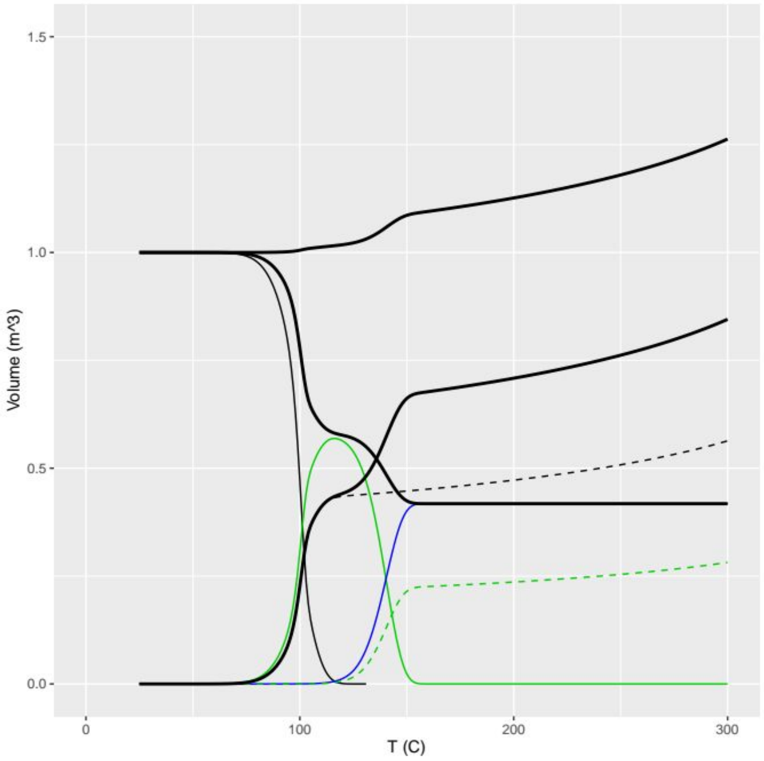

Figure S10: Volume of the different solids and fluids involved in the thermal decomposition of carnallite. The initial point is 1[3] of pure carnallite. Successive dehydration of this volume of hexa-hydrate (thin black

solid-line curve) produced a decreasing volume of the di-hydrate (thin green solid-line curve) and anhydrous salt (blue thin solid line), and the corresponding volume of water (dashed lines of the same color). The water volume increased even after the decomposition due to the reduction of water density with increasing temperature. The thick solid lines show total volumes (for solids, fluids and total).

2.2. The impact of the hydrolysis reactions

As discussed above, up to 200 ºC the total volume increases as water accumulates, and causes a volume increase of almost 25%. For higher temperatures, the dominant reaction is the hydrolysis of the mono-hydrate, that releases HCl, but not water. So, basically, the system behaves as a source of water up to 200 °C and a source of HCl for T>200 ºC. The effect of HCl release on total volume is not included in **Fig. S9**, because density data for HCl saturated solutions at these P/T conditions temperatures is not available. **Figure S11** compares the density of water at 21.2 MPa (the computed hydrostatic pressure) and the corresponding temperature and density of a solution at atmospheric pressure and at the same temperature. Data for these solutions at atmospheric pressure are tabulated for concentration values from 0 to saturation in ref[15]. These data were fitted as linear functions of the concentration and temperature and extrapolated to the 25<T<500ºC range. **Figure S12** shows the concentration of a hypothetical solution produced by dissolving the temperature-dependent HCl yield by hydrolysis reactions into the water produced by the previous dehydration reactions. The final concentration is slightly higher than the solubility at room temperature and pressure (around 38%), so probably some HCl is present as gas, but solubility at the simulated conditions is not known, so the presence of this gas is hypothetical. In any case, solutions with concentration close to 40% have negative pH values close to -1 or -2 at room temperature.

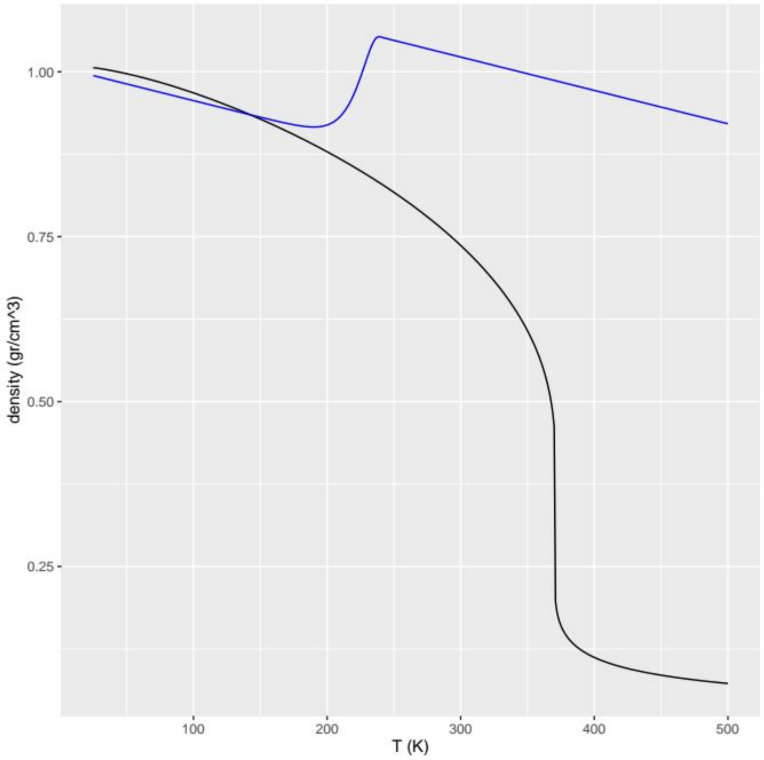

Figure S11: Density of water (black) and a solution resulting from the dissolution of all the HCl released by hydrolysis reactions in all the H_2O released by dehydration reactions (blue). The solution density was computed at atmospheric pressure and from extrapolated data, so it is not fully reliable, especially for temperatures close to the water boiling point.

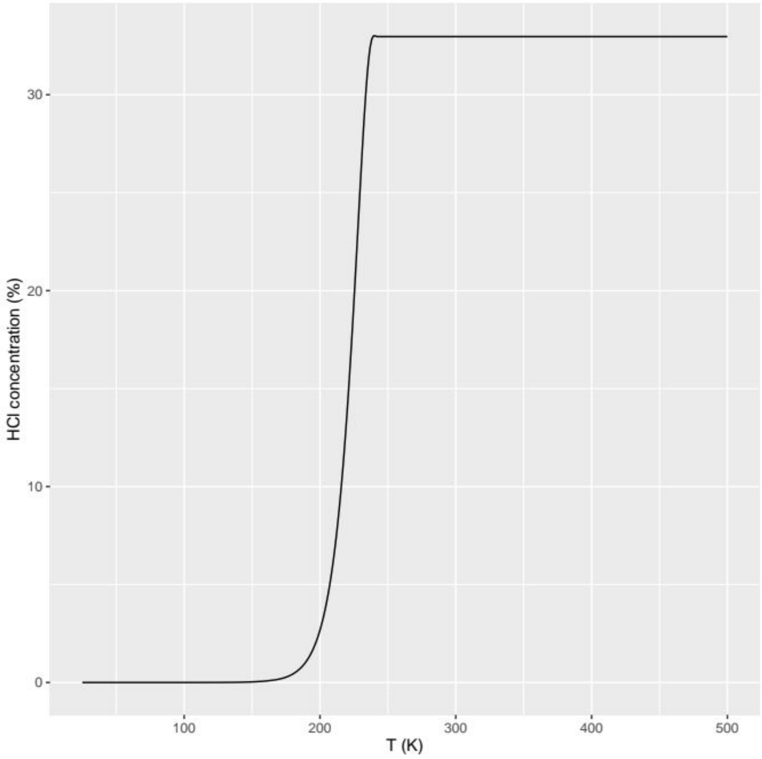

Figure S12: Concentration resulting from dissolving the HCl released by the hydrolysis of the mono-hydrate into the water accumulated from the dehydration of the 6, 4, 2 and 1 hydrates.

3. Thermal evolution of the Dallol area

The heat transfer equation describing the transient temperature field in the absence of a heat source in a lagrangian framework,

$$\rho c \frac{\partial T}{\partial t} = \nabla \cdot (k \nabla T) \qquad (1)$$

where T, t, ρ, c and k represent temperature, time, density, specific heat and thermal conductivity respectively, was numerically integrated using a finite difference algorithm with explicit Euler timestepping:

$$T_{i,j}^{n+1} = T_{i,j}^{n} + \Delta t \alpha \frac{T_{i+1,j}^{n} - 2T_{i,j}^{n} + T_{i-1,j}^{n}}{\Delta x^2} + \Delta t \alpha \frac{T_{i,j+1}^{n} - 2T_{i,j}^{n} + T_{i,j-1}^{n}}{\Delta y^2} \quad (2)$$

where

$$\alpha = \frac{k_{li}}{\rho_{li} c_{li}} \qquad (3)$$

is the lithology-dependent thermal diffusivity. The temperature dependence of k_{li} is implemented as

$k_{li} = \frac{k_0}{1 + bT}$ (4)

were b is an empiric parameter. Values for ρ_{li}, c_{li}, k_0 and b are listed for the involved lithologies in **Table S3** (adapted from ref[16]). **Figure S13** shows the resulting thermal diffusivities (α) used in the numerical calculation for each lithology and temperature.

Table S3: Parameters of the thermal model[16].

Litology	ρ (kg/m^3)	c (J/kg/K)	k0 (W/m/K)	b
Sandstone	2650	850	5.573	0.002600927
Limestone	2710	900	2.986	0.001785714
Shale	2690	900	1.935	0.000671296
Basalt	2850	800	2.200	0.0
Salt	2190	855	6.430	0.00483

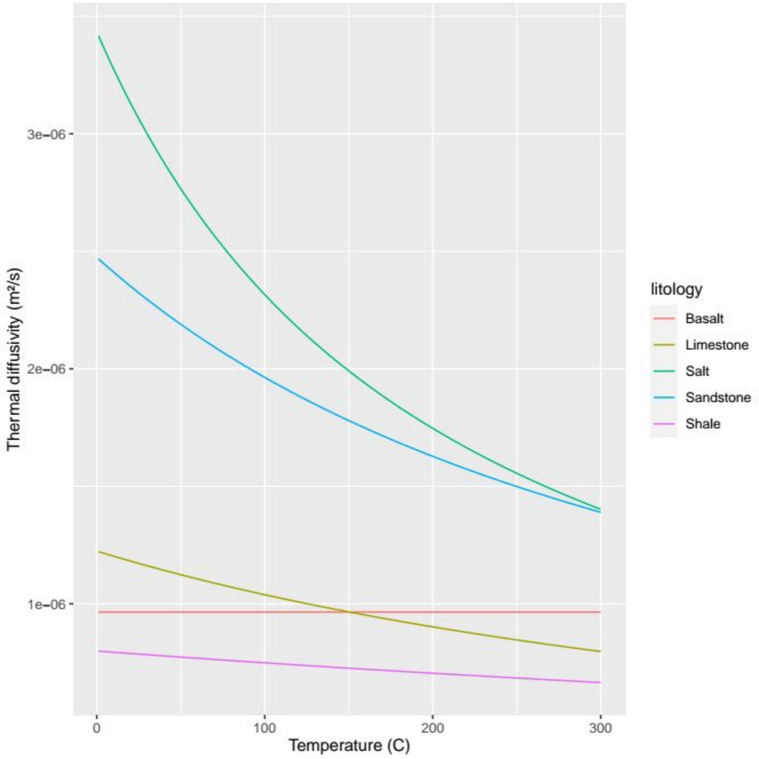

Figure S13: Thermal diffusivity as a function of temperature for the different lithology's used in the calculation of the transient gradient after the magmatic intrusion under Dallol (computed from equations 3 and 4 using the parameters in Table S3)

Discretized versions of **Fig. S7** were used to store the transient temperatures $T_{i,j}^{n}$ and as a lookup table to the k_0 values for the litology at cell i,j. **Figure S14** shows the array encoding the lithology during the calculations. **Figures S15** and **S16** show the k and T arrays by the end of the calculations (500000 years), using these arrays with $0 \leq i < 161$. $0 \leq j < 73$, $\Delta x = \Delta y = 100$m. The simulation, using Python[17] and numpy[18] to implement the explicit Euler time stepping algorithm, was run for 500000 years in 200000 steps ($\Delta t = 2.5$y). Each iteration involves the calculation of the new distribution of temperatures at each cell T(i,j) by the step() function with periodic boundaries implemented in the periodic boundary() function:

```
def periodic_boundary ():
  for y in range (Ny+2):
    f[0,y]=f[1,y]
    k0[0,y]=k0[1,y]
    b[0,y]=b[1,y]
    T[0,y]=T[1,y]
    f[Nx+1,y]=f[1,y]
    k0[Nx+1,y]=k0[1,y]
    b[Nx+1,y]=b[1,y]
    T[Nx+1,y]=T[1,y]
```

```
def step ():
  global k,time
  time+=dt
  k=f*k0/(1+b*T)
  dT = dt*( ( (k[1:Nx+1,1:Ny+1]+
               k[2:Nx+2,1:Ny+1])/2 *
              (T[2:Nx+2,1:Ny+1] - T[1:Nx+1,1:Ny+1])+
              (k[1:Nx+1,1:Ny+1]+
               k[0:Nx,1:Ny+1])/2 *
              (T[0:Nx,1:Ny+1] - T[1:Nx+1,1:Ny+1]) ) /
            dx**2 +
            ( (k[1:Nx+1,1:Ny+1]+
               k[1:Nx+1,2:Ny+2])/2 *
              (T[1:Nx+1,2:Ny+2] - T[1:Nx+1,1:Ny+1])+
              (k[1:Nx+1,1:Ny+1]+
               k[1:Nx+1,0:Ny])/2 *
              (T[1:Nx+1,0:Ny] - T[1:Nx+1,1:Ny+1]) ) /
            dy**2 )
  T[1:Nx+1,1:Ny+1]+=dT*tchange[1:Nx+1,1:Ny+1]
  periodic_boundary ()
```

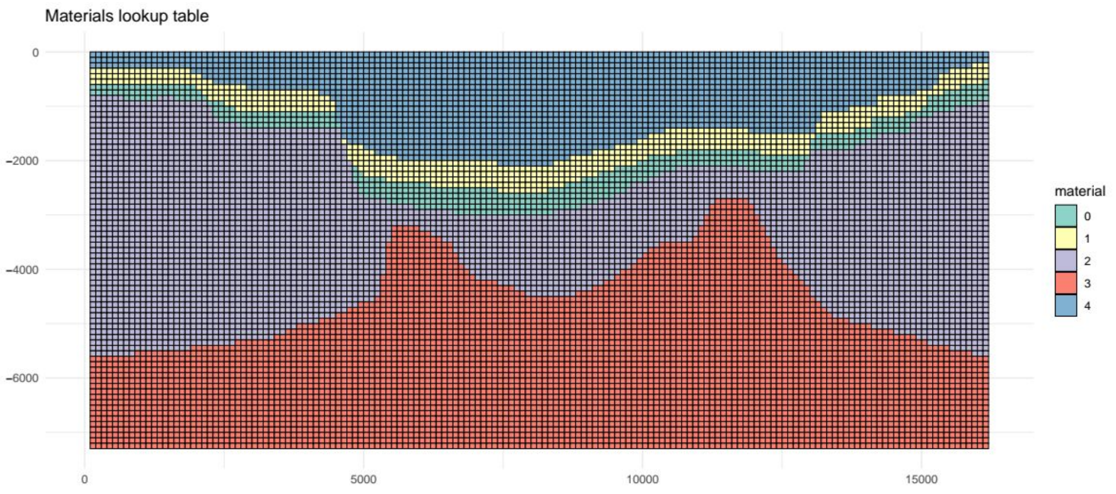

Figure S14: Representation of the array of integers encoding the lithology during calculations. 0=sandstone, 1=limestone, 2=shale, 3=basalt, 4=salt. This array is used as a lookup table to select the parameters of the model used for calculations in each cells from Table S3.

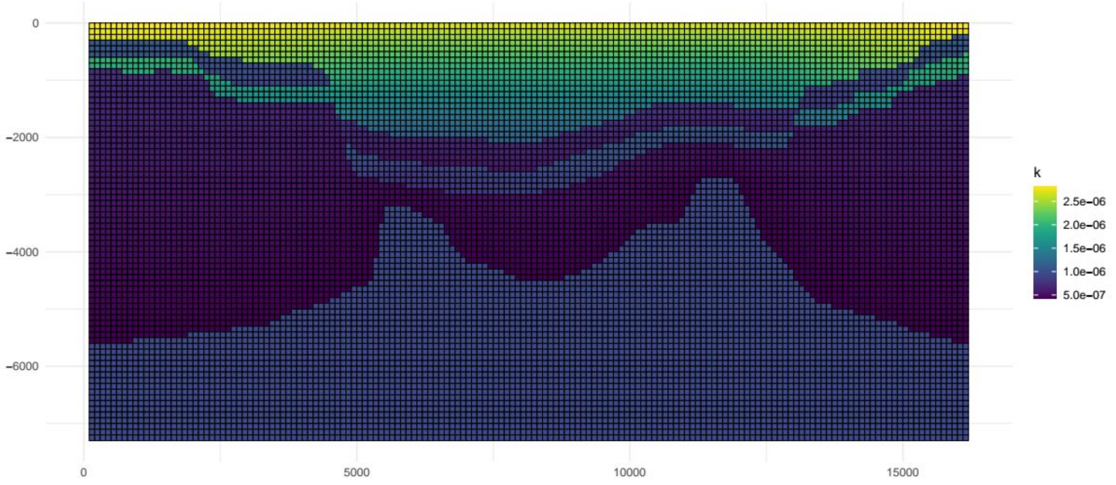

Figure S15: representation of the k-array computed from equation 3 from the lithology-dependent values of b and k_0, (see Table S3). Note that this value is not homogeneous within each lithology because it is temperature-dependent. These variations are particularly important within salt and sandstone.

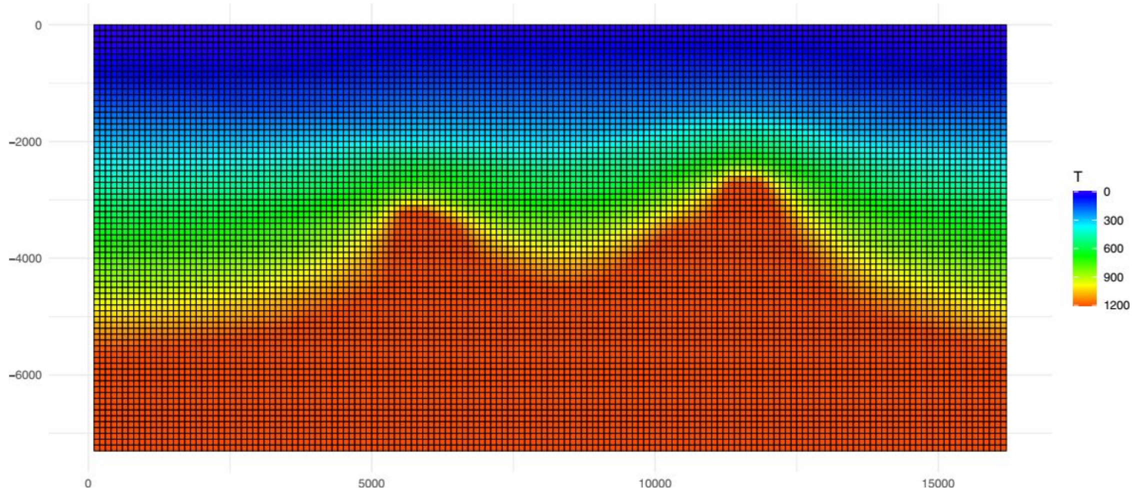

Figure S16: Representation of the discrete temperature array during the last iteration of the calculation (t=500000years).

During the calculation, each 100 iterations, the Temperature matrix **(Fig. S16)** is saved to disk and a plot like those in **Fig. 9** is generated for this time-step. These figures were created programmatically using R[19], using the ggplot2 graphical package[20], under the control of the calculation script that generates the corresponding R script and launches the application.

A video showing all the generated frames in sequence was encoded from the generated plots using the ffmpeg software[21], and can be downloaded as part of the supplementary material.

References

1. Mamani, V., Gutiérrez, A. & Ushak, S. Development of low-cost inorganic salt hydrate as a thermochemical energy storage material. *Sol. Energy Mater. Sol. Cells* **176**, 346–356 (2018).
2. *xyscan*. (Yale University).
3. *R Software*.
4. Lan, K. & Jorgenson, J. W. A hybrid of exponential and gaussian functions as a simple model of asymmetric chromatographic peaks. *J. Chromatogr. A* **915**, 1–13 (2001).
5. American Mineralogist Crystal Structure Database.
6. “MgOHCl (MgCl[OH], T = 898 K)” Crystal Structure: Datasheet from “PAULING FILE Multinaries Edition – 2012”.
7. Agron, P. A. & Busing, W. R. Magnesium dichloride hexahydrate, MgCl2.6H2O, by neutron diffraction. *Acta Crystallogr. C* **41**, 8–10 (1985).
8. Sugimoto, K., Dinnebier, R. E. & Hanson, J. C. Structures of three dehydration products of bischofite from *in situ* synchrotron powder diffraction data ($MgCl_2 \cdot nH_2O$; n = 1, 2, 4). *Acta Crystallogr. B* **63**, 235–242 (2007).
9. Wagner, W. & Pruß, A. The IAPWS Formulation 1995 for the Thermodynamic Properties of Ordinary Water Substance for General and Scientific Use. *J. Phys. Chem. Ref. Data* **31**, 387–535 (2002).
10. *IAPWS95*.

11. Schlemper, E. O., Sen Gupta, P. K. & Zoltai, T. Refinement of the structure of carnallite, Mg(H2O)6KCl3. *Am. Mineral.* **70**, 1309–1313 (1985).

12. Brynestad, J., Yakel, H. L. & Smith, G. P. Temperature Dependence of the Absorption Spectrum of Nickel(II)-Doped KMgCl $_3$ and the Crystal Structure of KMgCl $_3$. *J. Chem. Phys.* **45**, 4652–4664 (1966).

13. Fangh�nel, Th., Emons, H.-H. & K�hnke, K. Solid-liquid phase equilibria in the system KCl?MgCl2?H2O at elevated temperatures. III. Isotherms at 160, 170, and 180�C. *Z. F�r Anorg. Allg. Chem.* **576**, 99–107 (1989).

14. Boultif, A. & Louër, D. Powder pattern indexing with the dichotomy method. *J. Appl. Crystallogr.* **37**, 724–731 (2004).

15. Green, D. & Perry, R. "Perry's Chemical Engineers' Handbook" eighth edition. (2007).

16. Mello, U. T., Karner, G. D. & Anderson, R. N. Role of salt in restraining the maturation of subsalt source rocks. *Mar. Pet. Geol.* **12**, 697–716 (1995).

17. Van Rossum, G. *Python tutorial, Technical Report CS-R9526.* (1995).

18. Harris, C. R. *et al.* Array programming with NumPy. *Nature* **585**, 357–362 (2020).

19. R Core Team. *R: A language and environment for statistical computing.* (R Foundation for Statistical Computing, 2013).

20. Wickham, H. *ggplot2: Elegant Graphics for Data Analysis*. (Springer International Publishing : Imprint: Springer, 2016). doi:10.1007/978-3-319-24277-4.

21. Tomar, S. Converting video formats with FFmpeg. *Linux J.* **146**, (2006).
